# Hen egg white bovine colostrum supplement reduces symptoms of mild/moderate COVID-19: a randomized control trial

**DOI:** 10.2144/fsoa-2023-0024

**Published:** 2023-07-20

**Authors:** Jaclyn Kelly Mann, Tarylee Reddy, Mary van der Stok, Ayanda Ngubane, Takalani Mulaudzi, Nobuhle Mchunu, Portia Nevhungoni, Nithendra Manickchund, Pariva Manickchund, Chelline Helena Louise Cairns, Vaneshree Govender, Thumbi Ndung'u, Mahomed Yunus Suleman Moosa, Bernadett Isabel Gosnell

**Affiliations:** 1 HIV Pathogenesis Programme, University of KwaZulu-Natal, Durban, 4001, South Africa; 2 Biostatistics Research Unit, South African Medical Research Council, Durban, 4091, South Africa; 3 Department of Infectious Diseases, University of KwaZulu-Natal, Durban, 4001, South Africa; 4 Aurum Institute, Johannesburg, 2194, South Africa; 5 Africa Health Research Institute, Durban, 4001, South Africa; 6 Ragon Institute of Massachusetts General Hospital, Massachusetts Institute of Technology & Harvard University, Cambridge, MA 02139, USA; 7 Division of Infection & Immunity, University College London, London, WC1E 6BT, UK

**Keywords:** colostrum, coronavirus, COVID-19, delta, egg white, lactoferrin, lysozyme, nutritional supplement, omicron, ovotransferrin

## Abstract

**Aim:**

The ability of a hen egg white bovine colostrum supplement to prevent severe COVID-19 was tested in a double-blind randomized control study.

**Methods:**

Adults with mild/moderate COVID-19, risk factors for severe disease, and within 5 days of symptom onset were assigned to the intervention (n = 77) or placebo (n = 79) arms. Symptoms were documented until day 42 post-enrollment and viral clearance was assessed at 11–13 days post-symptom onset.

**Results:**

One participant developed severe COVID-19. The severe-type symptom score was lower in the active arm at 11–13 days post-symptom onset (p = 0.049). Chest pain, fever/chills, joint pain/malaise, and sore throat were significantly less frequent in the active arm. No differences in viral clearance were observed.

**Conclusion:**

The intervention reduced symptoms of mild/moderate COVID-19.

**Clinical Trial Registration:**

DOH-27-062021-9191 (South African National Clinical Trials Register)

## Plain language summary

Natural proteins found in milk (lactoferrin) and egg white (ovotransferrin and lysozyme) could have therapeutic value in COVID-19 through their effects on the immune system. We identified bovine colostrum and hen egg white powders containing adequate quantities of these proteins. We investigated whether short-term daily consumption of a hen egg white and bovine colostrum mixture (reconstituted with glycerin and water) could reduce the risk of progression to severe disease and assist in the recovery of patients with mild or moderate COVID-19. Adults with mild or moderate COVID-19 who were within 5 days of symptom onset and had risk factors for severe disease were enrolled, and randomly assigned to take a hen egg white and bovine colostrum mixture or placebo mixture twice daily for 5 days, and then followed up telephonically for 6 weeks. The main findings were that consumption of the hen egg white and bovine colostrum mixture was associated with fewer protocol-defined severe-type symptoms overall, and in particular lower frequencies of joint pain/malaise, chest pain, fever/chills, and sore throat. Only one individual developed severe COVID-19 and therefore the effect of the intervention on reducing the risk of progression to severe disease could not be assessed. The results of this study suggest that consumption of the hen egg white bovine colostrum mixture within a few days of symptom onset lessens symptoms in people with mild or moderate COVID-19.

## Tweetable abstract

Consumption of a hen egg white bovine colostrum mixture within a few days of symptom onset lessened symptoms in people with mild or moderate COVID-19.

## Graphical abstract

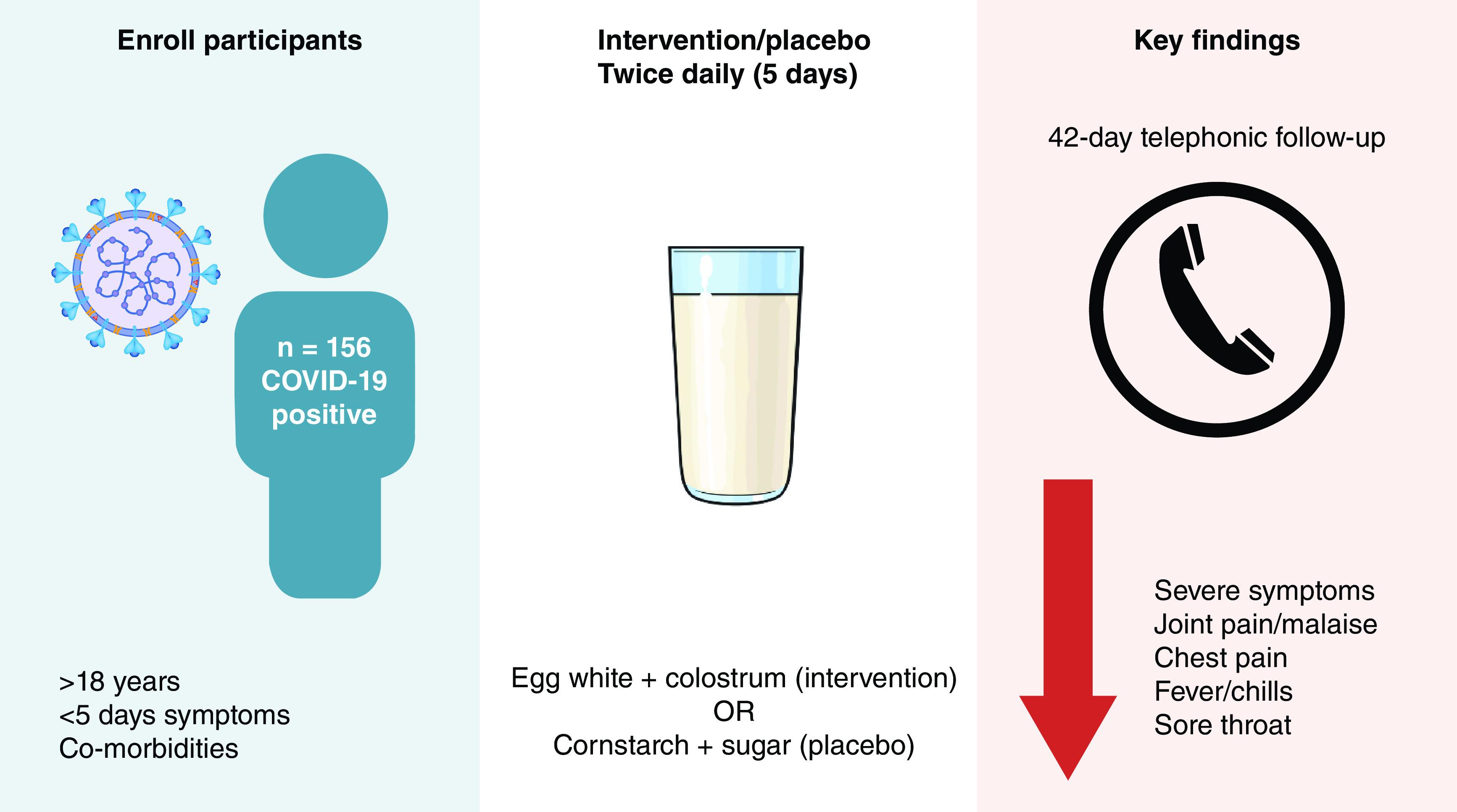

The COVID-19 pandemic, caused by SARS-CoV-2, has spread rapidly, with over 600 million infections resulting in over 6 million deaths worldwide [1]. The majority of symptomatic COVID-19 cases are mild to moderate, however a minority experience excessive inflammatory responses [2]. Further, a substantial proportion of infected individuals develop long COVID-19 [3]. While COVID-19 vaccines are available, the effectiveness of these may be undermined by escape variants and may wane over time [4]. Approved treatments for COVID-19, such as monoclonal antibodies and antivirals (e.g., remdesivir, molnupiravir, and paxlovid), remain prohibitively priced and largely unavailable in resource-limited settings, and some have unknown risks [5]. Readily available, safe, inexpensive treatments would have value in resource-limited settings.

Severe COVID-19 disease is characterized by disruption in iron homeostasis, oxidative stress, excessive pro-inflammatory cytokine production, pathological renin-angiotensin-system activation, and thrombus formation [6]. It is hypothesized that the natural proteins lactoferrin, ovotransferrin, and lysozyme, which are abundant in nature and have wide-ranging antiviral as well as immunomodulatory properties [7–10], may mitigate COVID-19-associated pathology. Lactoferrin may restore iron homeostasis through sequestering free iron and modulating proteins involved in balancing iron between blood and tissue [11]. Bovine lactoferrin has also been shown to potently inhibit SARS-CoV-2 replication *in vitro* [12]. Ovotransferrin has antiviral peptides, as well as iron-regulating and immunomodulatory activities, that are similar to that of human lactoferrin and bovine lactoferrin [13,14]. Both lactoferrin and lysozyme have been described as “immune sensing” with the ability to stimulate immune responses or dampen inflammation depending on circumstances [9,13]. Lactoferrin, ovotransferrin, and lysozyme exhibit antioxidant [9,13,15–17], anti-inflammatory [18–21], anti-thrombotic [22], and/or renin-angiotensin-system inhibitory [13,20,23–25] activities. Human clinical trials and/or animal model studies of lactoferrin, lysozyme, and ovotransferrin peptides have shown a significant reduction in the severity or incidence of viral diseases, with reduced viral loads in some instances [20,26–29]. Benefit has also been demonstrated in a range of diseases associated with immune pathology and iron dysregulation [11,18,30–36]. In the majority of studies, bovine lactoferrin and hen egg white lysozyme were administered orally and showed beneficial systemic effects as well as excellent safety profiles.

Bovine lactoferrin is present in high concentrations in colostrum, while ovotransferrin and lysozyme are abundant in hen egg white. Additional peptides in colostrum and egg white may support the activities of these proteins [13,17]. We therefore hypothesized that ingestion of good quality commercially available bovine colostrum powder and egg white powder, containing largely intact lactoferrin, ovotransferrin, and lysozyme in adequate quantities, could inhibit the progression of mild or moderate COVID-19 to severe disease and assist in recovery. We tested this hypothesis in patients with mild or moderate COVID-19 who were at increased risk of severe disease and were within 5 days of symptom onset at the time of enrollment.

## Materials & methods

### Ethics

The protocol was approved by the Biomedical Research Ethics Committee of the University of KwaZulu-Natal (BREC 00002220/2020) and the KwaZulu-Natal Department of Health (KZ_202103_026). The trial was registered with the South African National Clinical Trials Register (DOH-27-062021-9191). Written informed consent was obtained from all participants.

### Study design, setting & participants

The study was a double-blind randomized control trial with 6 weeks follow-up of participants. It was conducted in Durban, in the KwaZulu-Natal province of South Africa, from 28 July 2021 to 5 July 2022. The trial was stopped before reaching the targeted number of participants due to a combination of factors. These included a marked reduction in the number of patients presenting for testing due to a better understanding of the natural history of the disease by both the public and healthcare workers, limitation in study funds, and the investigational product reaching its expiry date. Participants were recruited from King Edward VIII Hospital and Cato Manor Clinic. Individuals underwent the screening process if they were likely to meet the study criteria and were willing to be screened. Eligible participants were adults (i.e., ≥18 years of age) with a positive rapid SARS-CoV-2 antigen test result who were able to provide informed consent, were within 5 days of onset of mild or moderate symptoms compatible with COVID-19 illness, were available and contactable for the 6 weeks of follow-up, and were at increased risk of severe COVID-19. Risk factors for severe disease included age >50 years and/or one of the following co-morbidities (using reference [37] as a guide): cardiovascular disease, chronic obstructive pulmonary disease, uncontrolled asthma, chronic lung disease, diabetes, immunocompromise (due to tuberculosis, human immunodeficiency virus - HIV, or cancer), end-stage renal disease requiring dialysis, liver disease or obesity with body mass index (BMI) >35. The list of risk factors in the inclusion criteria was broadened on 25 November 2021 based on updated literature and to facilitate improvement in recruitment. Uncontrolled asthma was changed to asthma [38], end-stage renal disease requiring dialysis was changed to chronic kidney disease [39], obesity with BMI >35 was changed to overweight/obese with BMI ≥25 [40–42], and additional risk factors were included, namely, pregnancy [43], mental illness (e.g., anxiety, depression, substance misuse, psychosis, schizophrenia) [44,45] and neurological disorders (e.g., Alzheimer's disease, Parkinson's disease, and epilepsy) [46]. Exclusion criteria were allergy or contraindication (e.g., lactose intolerance) or objection (e.g., religious) to the study product components, severe or asymptomatic COVID-19 at baseline, a negative/inconclusive SARS-CoV-2 test result, no access to a mobile phone, participation in other clinical trials with investigational agents within 8 weeks before study start, or any condition for which participation in the study, as judged by the investigator, could compromise the well-being of the subject or prevent, limit or confound protocol-specified assessments.

Participants were randomized in a 1:1 ratio into the active or placebo arm using a block randomization sequence, with randomly selected block sizes of 4, 6, and 8 matched to participant identifiers, which was prepared by the unblinded statistician. For the purposes of blinding participants and all study staff involved in patient care and data collection, the unblinded study statistician generated 5 unique codes that represented the placebo and another 5 unique codes that represented the active product and provided these to the packaging company for printing on the outer labels of the products. The unique codes were matched to participant identifiers by the statistician, and the use of this randomization schedule enabled the correct allocation of study products to the participants by the nurses.

At enrollment (day 0), information on the following was collected: demographics, medical history, dietary history, vital signs (including respiratory rate, oxygen saturation [SpO_2_], pulse, temperature, and blood pressure), symptoms, functional status (as an indicator of well-being), and concomitant medications. Dietary history included information on egg and milk consumption. Naso- and oro-pharyngeal swabs were both collected, combined in a single tube, and sent for confirmatory real-time SARS-CoV-2 reverse transcriptase polymerase chain reaction (RT-PCR) testing at the neighboring African Health Research Institute clinical laboratory as per the guidelines from the National Institute for Communicable Diseases in South Africa. At enrollment, a follow-up clinic visit was scheduled for 11–13 days post-symptom onset. Following enrollment, individuals who did not require hospital admission as part of the standard of care either returned home to isolate for the period recommended by national guidelines or were referred to a step-down facility for isolation if they could not safely isolate at home. On days 1–5 post-enrollment, information on study product adherence was collected telephonically and used to calculate the total number of doses of study product taken. The participants also returned their empty sachets at the follow-up clinic visit (11–13 days post-symptom onset) as an additional measure of adherence. Adherence was defined as taking 80% or more of doses as measured by either the study product intake questionnaire or the empty sachet return count. At the follow-up clinic visit, naso- and oro-pharyngeal swabs were both collected, combined, and tested at the Africa Health Research Institute clinical laboratory for SARS-CoV-2 using the real-time SARS-CoV-2 RT-PCR test. Information on vitals, symptoms, functional status, and concomitant medications was also collected at the follow-up clinic visit. On days 2, 4, 7, 14, and 28 post-enrollment, information on symptoms, functional status, and concomitant medications was collected via telephonic follow-up. On day 42 post-enrollment, information was collected telephonically on post-COVID symptoms and a study completion form was completed. If a participant was not successfully contacted/seen within the protocol-defined acceptable window for a particular visit, then that constituted a missed visit, however, contact was still attempted for each visit thereafter up to day 42.

Any new symptoms not present at enrollment, worsening of symptoms/conditions, or hospitalization (all captured on the symptom questionnaires), whether related to COVID-19 illness or otherwise, were documented as adverse events and a judgment on the relatedness of the adverse event to study products was made by the study clinicians. The severity of adverse events was graded using the Division of AIDS Table for Grading the Severity of Adult and Pediatric Adverse Events, Version 2.1, July 2017. Adverse events grade 3 and higher were severe adverse events and these were reported to the ethics committee and were also reviewed by the data and safety monitoring committee.

### Study products

#### Composition & form

**Figure 1.**
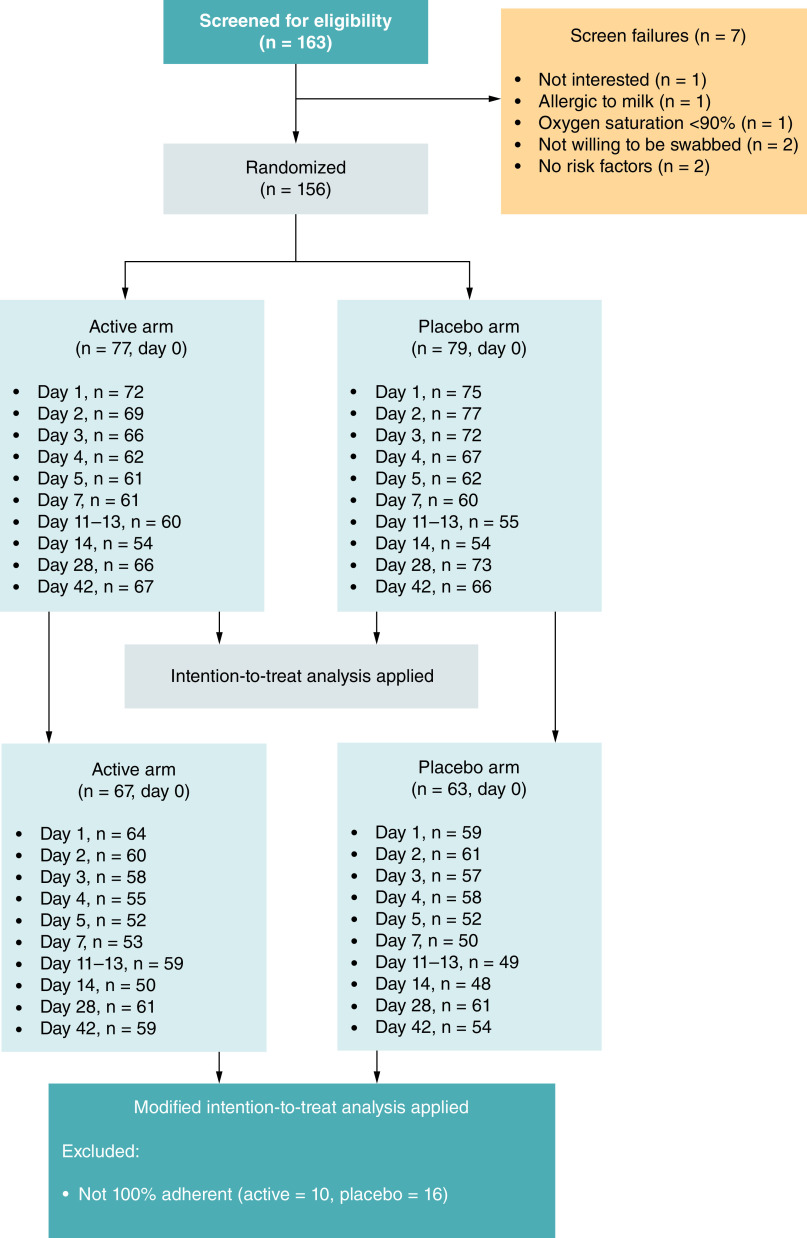
The number of individuals with COVID-19 who were screened, enrolled, and analyzed. Individuals at outpatient clinics underwent the screening process if they received a positive rapid SARS-CoV-2 antigen test result and were likely to meet the study criteria. In total, 163 individuals were screened and 156 were enrolled, where 79 and 77 were randomized to the placebo and active arms, respectively. The number of participants who were contactable/seen within the visit window for each visit (days 0, 1–5, 7, 14, 28, and 42 post-enrollment and 11–13 days post-symptom onset) are shown. All of these individuals were included in the intention-to-treat analysis. The subset included in the modified intention-to-treat analysis (those participants who took all doses of the study product) is shown for each visit in both arms.

The active study product was egg white powder supplement (EAP sport plus, Pulviver, distributed by A&D Food Ingredients, South Africa) and colostrum milk powder (bovine colostrum powder, Biolac SA, South Africa), reconstituted using glycerin meeting the requirements of British Pharmocopoeia (glycerin BP; Organic Chemical Corporation, South Africa) and distilled water (EasiHealth Nutraceuticals, South Africa). Egg white (12 g) and colostrum (20 g) powders were blended and packaged in a sachet, and glycerin BP (12 g) and distilled water (22 g) were packaged in another sachet. One powder sachet reconstituted with one liquid sachet constituted one dose. The particular egg white and colostrum products chosen were based on tests measuring undenatured/intact forms of lysozyme, ovotransferrin, and lactoferrin (Supplementary Table 1 & Figure 1). The quantities of egg white and colostrum were chosen based on quantities of lactoferrin (100 mg per day [20]) and lysozyme (60 mg–1 g per day [10,28,29]) previously shown to have clinical benefit in viral or inflammatory diseases following ingestion. The daily amount of egg white powder and colostrum powder (in 2 divided amounts) was 24 g egg white (containing ≈ 0.78 g lysozyme and ≈ 2.65 g ovotransferrin) and 40 g colostrum powder (containing ≈ 100 mg lactoferrin). Briefly, one sachet of egg white and colostrum contained approximately 631 kj energy, 16.14 g protein, 5.44 g carbohydrate, 6.7 g fat, and 0.6 g fiber.

The placebo study product, designed to closely match the active study product in appearance, was comprised of cornstarch (Belville Mill, South Africa), icing sugar (RCL Foods, South Africa), and egg yellow powder (Osman's Spice Works, South Africa), reconstituted with distilled water. Cornstarch (16 g), icing sugar (8 g), and egg yellow powder (0.0016 g) were blended and packaged in a sachet and were reconstituted with distilled water (40 g) that was packaged in another sachet. Briefly, one sachet of the placebo mix contained approximately 22.6 g carbohydrate and 385 kj energy.

Study products were stored at room temperature, protected from direct sunlight.

#### Administration

The active and placebo study products were administered in the same manner. Immediately following randomization, a package of the 10 doses of study product was provided to the participant with verbal explanation and written instructions in the participant's preferred language. Immediately before ingestion, one powder sachet was mixed with one liquid sachet and allowed to reconstitute for 5 minutes. The first dose was taken by the patient under the supervision of a nurse. The nurse observed the participant for any immediate adverse effects while the remaining enrollment visit procedures were carried out. The participant was instructed to take the remaining 9 doses on an empty stomach twice daily (beginning on the evening of day 0) - once in the morning on waking up (at least 1 hour before eating) and once in the evening before sleeping (at least 2 hours after eating). The participant was reminded to keep all their empty sachets in the Ziploc bag provided and return them when coming in for the follow-up clinic visit.

### Primary outcome

The primary outcome of the study was progression to severe disease as defined by the World Health Organization (clinical signs of pneumonia [fever, cough, dyspnea, fast breathing] plus one of the following: respiratory rate >30 breaths/min; severe respiratory distress; or SpO_2_ <90% on room air [47]) by 11–13 days post-symptom onset and within 28 days post-enrollment. Information on clinical symptoms was collected telephonically (on day 0, as well as on days 2, 4, 7, 14, and 28 after enrollment) as well as at the follow-up clinic visit (11–13 days post-symptom onset). Hospitalization since the last visit was also documented on the clinical symptom questionnaire. If hospitalization occurred, respiratory rate and SpO_2_ were obtained from hospital records to assess, together with clinical symptoms, whether or not an individual had progressed to severe disease. Respiratory rate and SpO_2_ were also measured at the follow-up clinic visit.

### Secondary outcomes

#### Symptom frequency & duration

The presence or absence of different individual symptoms was collected telephonically (on day 0, as well as on days 2, 4, 7, 14, and 28 after enrollment) as well as at the follow-up clinic visit (11–13 days post-symptom onset), and included fever/chills, shortness of breath, chest pain, palpitations, joint pain/malaise, sore throat, cough, fatigue, diarrhea/vomiting, anosmia, dysgeusia, headache, muscular weakness, anxiety/depression, sleep disturbances, cognitive disturbances (brain fog) and hair loss. Of these symptoms, fever/chills, shortness of breath, chest pain, palpitations, and joint pain/malaise were categorized as severe-type symptoms [48] while the remaining symptoms were categorized as non-severe. Individual symptom frequency at each visit was compared between study arms. The information on individual symptoms was also used to calculate a composite total symptom score (the sum of individual symptoms, i.e., the overall symptom frequency), composite severe symptom score, and composite non-severe symptom score for each visit. Change in symptom score from baseline was also calculated for each visit. The overall symptom duration was the time from symptom onset to the first visit at which the participant was symptom-free.

#### Karnofsky score

A Karnofsky score questionnaire [49], which measures functional status, was used as an indicator of well-being on day 0, as well as on days 2, 4, 7, 14, 28, and 42 post-enrollment and 11–13 days post-symptom onset.

#### Hospital admission

Hospital admission since the last visit was captured on days 2, 4, 7, 14, and 28 post-enrollment using the clinical symptom questionnaire. This end point pertained specifically to hospitalization by day 28 as a result of illness due to, or potentially exacerbated by, COVID-19.

#### Viral clearance

At the follow-up clinic visit (11–13 days post-symptom onset), naso- and oro-pharyngeal swabs were both collected, combined, and tested for the presence of SARS-CoV-2 using the real-time SARS-CoV-2 RT-PCR test. Results were categorized as “detected”, “not detected”, “inconclusive”, or “invalid”. The test was repeated with a stored remnant of the sample if the result was “invalid” or “inconclusive”. A “not detected” result (cycle threshold above 40) was considered to represent viral clearance.

#### Post-COVID-19 sequelae

Post-COVID-19 sequelae were defined as persisting or new symptoms present more than 4 weeks after the onset of acute symptoms of COVID-19 [3]. On day 42 after enrollment, a questionnaire was administered telephonically to document the presence or absence of symptoms (fatigue, muscular weakness, joint pain/malaise, shortness of breath, cough, anxiety/depression, sleep disturbances, anosmia, dysgeusia, cognitive disturbances, headaches, palpitations, chest pain, and hair loss) as well as the presence or absence of conditions (thromboembolism, diabetes, and chronic kidney disease) newly diagnosed or worsened since COVID-19 onset. The presence or absence of post-COVID sequelae, as well as symptom frequency, was compared between study arms.

### Statistical analyses

All analysis was performed using Stata 16.0. P-values <0.05 were considered statistically significant.

#### Sample size calculations

Sample size calculations were based on the reported rate of severe COVID-19 in symptomatic individuals in 2020 [47,50]. For the primary end point at 28 days, it was estimated that a minimum of 71 events, translating to a sample size of 240 patients per group, was required to detect at least 50% treatment efficacy with 80% power, where the clinical end point rate (severe disease) was 15%. To account for a 20% loss to follow-up, it was estimated that a total of 300 patients per arm was required to analyze the primary end point.

#### Baseline variables

Baseline demographic, dietary (egg and milk consumption), concomitant medication, and clinical data were summarized using means and standard deviations. Binary and categorical variables were presented using counts and percentages. All baseline characteristics were stratified by the treatment arm.

#### Analysis of end points

All end point analysis was performed on the intention-to-treat (ITT) principle, which included all randomized participants according to the treatment that they were randomized to. The modified intention-to-treat (mITT) analysis included only those randomized participants who took all doses of the study product. The primary end point could not be analyzed as there was only 1 event.

Individual symptom frequency at each visit was presented using descriptive statistics and compared between arms using the Chi-square test. Composite severe symptom scores, composite non-severe symptom scores, and composite total symptom scores were presented as means and standard deviations at each visit and compared between the arms using the unpaired *T*-test. Change in symptom frequency was calculated relative to the enrollment visit for each individual at each follow-up visit and compared between the arms using the unpaired *T*-test. The time to symptom resolution (for any symptoms and severe symptoms) was stratified by the treatment arm and analyzed using the Kaplan-Meier log-rank test for only those individuals with complete visits up to day 28. Individuals who were not symptom-free at the day 28 visit were censored at that time point.

Karnofsky scores at each visit were summarized using means and standard deviations and were compared between arms using the unpaired *T*-test, separately for each visit. Hospital admission by 28 days was summarized as counts and percentages by treatment arm. The proportion of participants with viral clearance (detected vs not detected) at 11–13 days post-symptom onset was summarized using counts and percentages and compared between study arms using the Chi-square test. Individual post-COVID-19 sequelae, as well as the presence of any post-COVID-19 sequelae, were summarized using counts and percentages, stratified by study arm. The Chi-square test was used to test for differences in the proportion of post-COVID-19 sequelae between study arms and the unpaired *T*-test was used to test for differences between study arms in the mean number of post-COVID-19 symptoms.

## Results

### Participant characteristics

A total of 163 participants were screened and 156 were enrolled, where 79 and 77 were randomized to the placebo and active arms, respectively (Figure 1). The number of participants who were contactable/seen within the visit window for each visit is shown in Figure 1. The ITT analysis is presented and included all participants, while the mITT analysis only included a subset. The results of the mITT analysis were very similar to that of the ITT analysis. Deidentified individual participant data that underlie the results reported in the article, together with the trial protocol, are available in Mendeley data (https://data.mendeley.com/datasets/79wzs93pf3/1).

The baseline symptom onset timing, demographics, co-morbidities, milk and egg intake scores, concomitant medications, COVID-19 vaccination status, and clinical characteristics were similar between study arms, except for a significantly higher frequency of the cough symptom in the active arm (84% vs 67%) and a higher frequency of muscular weakness in the placebo arm (5% vs 0%) (Table 1). The most common (>10% of participants) concomitant medications taken within the first 7 days of enrollment included analgesics, vitamins (vitamin A [3% of participants], vitamin B [19%], vitamin C [24%], vitamin D [14%], and multivitamin or not specified [10%]), minerals (zinc [34%], ferrous sulfate [11%], and other [3%]), antibiotics (azithromycin [26%], co-amoxiclav or amoxicillin [24%], and other [2%]), anti-inflammatory drugs (non-steroidal anti-inflammatories [14%], and corticosteroids [6%]), and anti-histamines (allergex [13%] and other [2%]). Half of the participants (43 in the placebo arm and 35 in the active arm) had received a COVID-19 vaccine before enrollment and 4% received a dose of COVID-19 vaccine during study follow-up.

**Table 1.** Summary of baseline demographics, dietary and clinical information.

Variable^†^		Placebo (n = 79)	Active (n = 77)	Total	p-value^¶¶^
Symptom onset, n (%)	≤3 days	45 (57.0)	41 (53.2)	86 (55.1)	0.75
Age, n (%)	>50 years	23 (29.1)	26 (33.8)	49 (31.4)	0.61
Sex, n (%)	Female	59 (74.7)	54 (70.1)	113 (72.4)	0.59
Race, n (%)	Black	64 (81.0)	58 (76.3)	122 (78.7)	0.12
	White	8 (10.1)	10 (13.2)	18 (11.6)	
	Indian	6 (7.6)	2 (2.6)	8 (5.2)	
	More than one race	1 (1.3)	6 (7.9)	7 (4.5)	
Income, n (%)	≤R 10000 per month	53 (67.1)	52 (67.5)	105 (67.3)	0.94
Level of education, n (%)	Matric or lower	47 (59.5)	48 (63.2)	95 (61.3)	0.74
Healthcare sector, n (%)	Yes	26 (33.8)	21 (28.4)	47 (31.1)	0.49
COVID-19 vaccine, n (%)	Yes	43 (54.4)	35 (45.5)	78 (50)	0.34
Medications, n (%)^‡^	Analgesics	45 (57.0)	44 (57.1)	89 (57.1)	1.00
	Vitamins	32 (40.5)	40 (51.9)	72 (46.2)	0.20
	Minerals	28 (35.4)	35 (45.5)	63 (40.4)	0.25
	Antibiotics	27 (34.2)	27 (35.1)	54 (34.6)	1.00
	Anti-inflammatories	14 (17.7)	12 (15.6)	26 (16.7)	0.83
	Anti-histamines	11 (13.9)	13 (16.9)	24 (15.4)	0.66
Pregnant, n (%)^§^	Yes	5 (8.8)	9 (17.6)	14 (13.0)	0.25
Co-morbidities, n (%)^¶^	≤1^#^	30 (38.0)	31 (40.3)	61 (39.1)	0.87
	≥2	49 (62.0)	46 (59.7)	95 (60.9)	
	Body mass index ≥25 kg/m^2^	58 (73.4)	63 (81.8)	121 (77.6)	0.25
	Hypertension^††^	18 (22.8)	18 (23.4)	36 (23.1)	1.00
	Cardiac condition^††^	1 (1.3)	2 (2.6)	3 (1.9)	0.62
	Thromboembolism^††^	1 (1.3)	0 (0)	1 (0.6)	1.00
	Dyslipidemia^††^	0 (0)	2 (2.6)	2 (1.3)	0.24
	Diabetes^‡‡^	6 (7.6)	7 (9.1)	13 (8.3)	0.78
	Glucose >11.1 mmol/l‡‡	1 (1.3)	3 (3.9)	4 (2.6)	0.36
	HIV	31 (39.2)	22 (28.6)	53 (34.0)	0.18
	Tuberculosis	2 (2.5)	4 (5.2)	6 (3.8)	0.44
	Cancer	2 (2.5)	4 (5.2)	6 (3.8)	0.44
	Asthma	19 (24.1)	16 (20.8)	35 (22.4)	0.70
	Neurological disease^§§^	1 (1.3)	0 (0)	1 (0.6)	1.00
	Epilepsy^§§^	1 (1.3)	0 (0)	1 (0.6)	1.00
	Depression/anxiety	1 (1.3)	0 (0)	1 (0.6)	1.00
Karnofsky score, n (%)	Mild	76 (96.2)	75 (97.4)	151 (96.8)	1.00
	Moderate	2 (2.5)	2 (2.6)	4 (2.6)	
	Severe	1 (1.3)	0 (0)	1 (0.6)	
Symptoms, n (%)	Fever/chills	44 (55.7)	43 (55.8)	87 (55.8)	0.99
	Shortness of breath	12 (15.2)	12 (15.6)	24 (15.4)	0.95
	Joint pain/malaise	14 (17.7)	20 (26)	34 (21.8)	0.21
	Chest pain	15 (19)	14 (18.2)	29 (18.6)	0.90
	Palpitations	1 (1.3)	0 (0)	1 (0.6)	0.32
	Sore throat	34 (43)	34 (44.2)	68 (43.6)	0.89
	Cough	53 (67.1)	65 (84.4)	118 (75.6)	**0.01**
	Fatigue	22 (27.8)	23 (29.9)	45 (28.8)	0.78
	Diarrhea/vomiting	6 (7.6)	8 (10.4)	14 (9)	0.54
	Anosmia	8 (10.1)	6 (7.8)	14 (9)	0.61
	Dysgeusia	6 (7.6)	10 (13)	16 (10.3)	0.27
	Headache	39 (49.4)	38 (49.4)	77 (49.4)	1.00
	Muscular weakness	4 (5.1)	0 (0)	4 (2.6)	0.04
	Anxiety/depression	2 (2.5)	0 (0)	2 (1.3)	0.16
	Sleep disturbance	3 (3.8)	0 (0)	3 (1.9)	0.08
	Cognitive disturbance	1 (1.3)	1 (1.3)	2 (1.3)	0.99
	Hair loss	0 (0)	0 (0)	0 (0)	-
Severe symptom score		1.09 (0.82)	1.16 (0.95)	1.12 (0.88)	0.64
Total symptom score		3.34 (1.8)	3.56 (1.86)	3.45 (1.83)	0.46
Milk intake, cups/week		12.03 (4.71)	12.63 (6.09)	12.32 (5.42)	0.74
Egg intake, eggs/week		4.87 (2.85)	5.25 (2.62)	5.06 (2.58)	0.28
Age, years		42.41 (11.99)	44.29 (13.64)	43.33 (12.83)	0.49
Body mass index, kg/m^2^		30.81 (8.31)	31.80 (7.61)	31.30 (7.96)	0.44
Mid-upper arm circumference, cm		32.45 (5.94)	33.09 (5.40)	32.77 (5.66)	0.35
Random glucose level, mmol/l		5.47 (1.94)	5.77 (2.16)	5.62 (2.05)	0.36
Systolic blood pressure, mmHg		123.19 (18.7)	124.47 (18.4)	123.83 (18.52)	0.94
Diastolic blood pressure, mmHg		82.56 (14.45)	82.88 (13.98)	82.72 (14.17)	0.82
Respiratory rate, breaths/minute		21.25 (1.64)	21.48 (1.85)	21.37 (1.75)	0.42
Oxygen saturation, percentage		98.73 (0.78)	98.56 (0.98)	98.65 (0.89)	0.22

† The mean and standard deviation are reported for continuous measures, while number and percentage (n [%]) are indicated for categorical variables.

‡ Concomitant medications/vitamins reported for ≥10% of participants within the first week of enrollment. Chronic medications taken for comorbid conditions are not listed.

§ Including women only.

¶ Cerebrovascular accident, chronic kidney disease, chronic obstructive pulmonary disease, chronic liver disease, and other mental health conditions were not reported by any participants.

# 4 individuals did not have any co-morbidities.

†† The presence of hypertension, cardiac condition, thromboembolism, dyslipidemia, and/or cerebrovascular accident were counted as one co-morbidity, namely cardiovascular disease.

‡‡ Previously diagnosed diabetes and/or a random glucose measurement of >11.1 mmol/l together with symptoms of unexplained weight loss or night-time waking to urinate were counted as one co-morbidity.

§§ Neurological disease and/or epilepsy were counted as one co-morbidity.

¶¶ P-values are from the unpaired *T*-test for continuous measures and the Chi-square test for categorical variables.

COVID-19 – coronavirus disease of 2019.

### Study product adherence

Adherence, defined as taking 80% or more of study product doses, was similar between the placebo and active arms (90% and 94%, respectively).

### Adverse events, severe COVID-19, & hospitalizations

There was a total of 131 adverse events (71 in the placebo arm and 60 in the active arm) in 84 different individuals (44 in the placebo arm and 40 in the active arm), occurring at a median of 3 days after enrollment (interquartile range, 2–7 days). The most common adverse events were new COVID-19-related symptoms (n = 66), long COVID-19 (n = 24), and gastrointestinal upset (n = 16). Nine adverse events (6 and 3 in the placebo and active arms, respectively) of mild/moderate gastrointestinal upset were deemed as possibly related to the study product. None of the adverse events that were ≥grade 3 were related to the study product.

A total of 6 individuals (4%) were hospitalized, as a result of illness either related to COVID-19 or other pre-existing conditions potentially exacerbated by COVID-19, during study follow-up. There was no difference in the frequency of hospitalization between the study arms (risk difference [95% confidence intervals]; RD [95% CI] = -2 [-8; 4]; p = 0.42). Four hospitalizations were in the placebo arm (placebo = 5.1% hospitalized; 1 with newly diagnosed tuberculosis and progression of HIV disease [HIV infection diagnosed before enrollment], 1 with newly diagnosed tuberculosis and advanced HIV disease, 1 with COVID-19 pneumonia with cardiomyopathy and 1 with COVID-19-related chest pain). Two hospitalizations were in the active arm (active = 2.6% hospitalized; 1 with multidrug-resistant tuberculosis [tuberculosis diagnosed before enrollment] and 1 with severe COVID-19). The latter met the definition of severe COVID-19 and progressed to respiratory failure and death. Of note, this participant was subsequently found not to meet inclusion criteria as they were enrolled >5 days after symptom onset.

### Symptom frequency & duration

Frequencies of individual symptoms, mean symptom scores, and mean changes in symptom scores are stratified by study arm and visit (with p-values, effect sizes, and confidence intervals) in Supplementary Tables 2–5. As expected, the frequency of symptoms declined in both study arms from day 0 to day 28.

**Figure 2.**
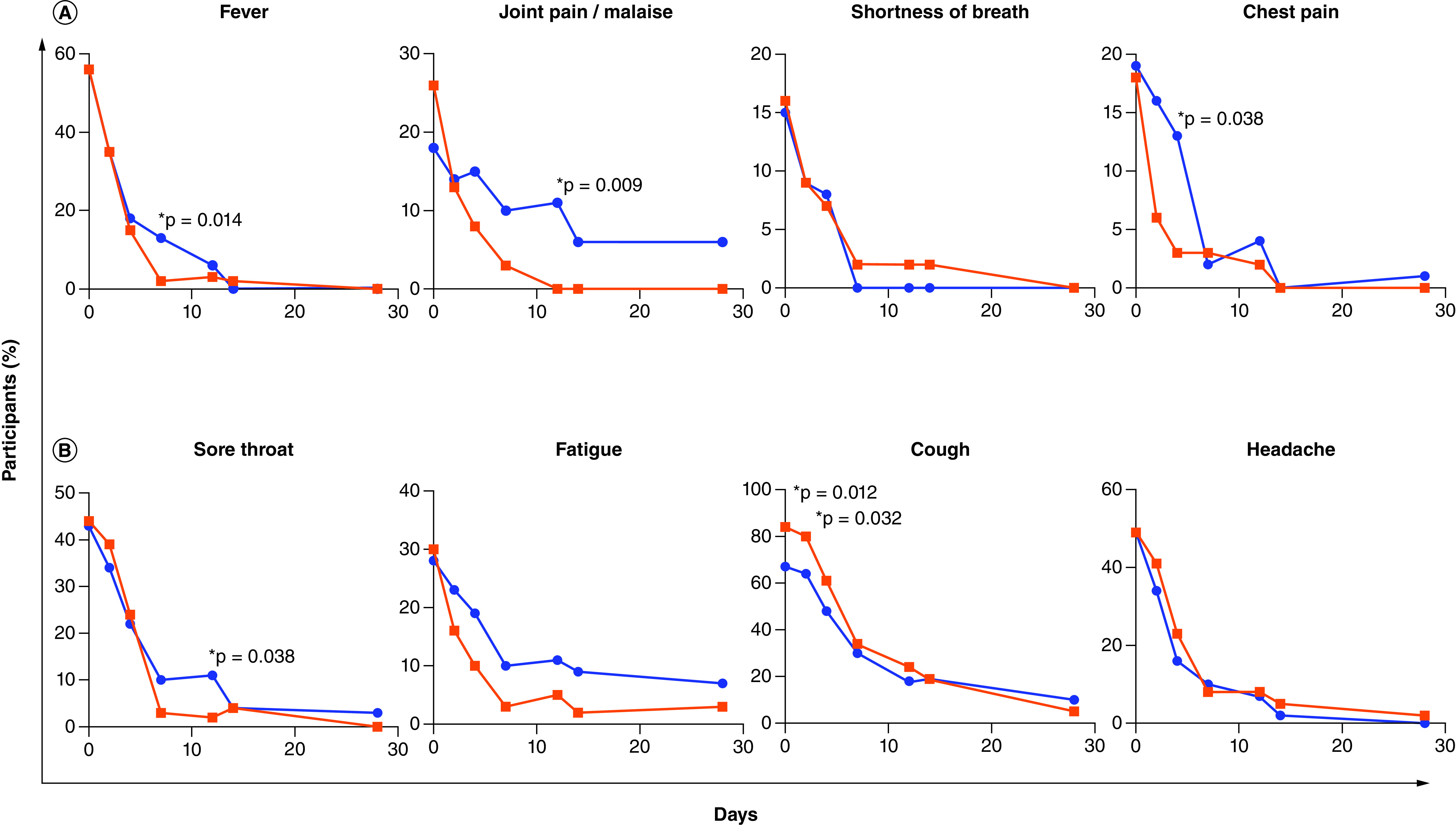
Symptom frequency. The percentage of participants with individual protocol-defined **(A)** severe-type and **(B)** non-severe-type symptoms is shown per study arm (placebo arm in blue and active arm in red) for symptoms present in more than 10% of individuals at enrollment. The Chi-square test was used to compare symptom frequency between study arms at each visit (days 0, 1–5, 7, 14, 28, and 42 post-enrollment and 11–13 days post-symptom onset) and p-values <0.05 are shown with an asterisk. The total number of participants in each arm at each visit is the same as that shown in Figure 1 (intention-to-treat analysis).

Individual symptoms present in more than 10% of individuals at enrollment (Table 1) included fever/chills, shortness of breath, joint pain/malaise, and chest pain in the severe symptom category, and sore throat, cough, fatigue, and headache in the non-severe symptom category (Figure 2). Individual severe-type symptoms with significantly lower frequency in the active arm were fever/chills (RD [95% CI] = -12 [-21; -3]; p = 0.014, day 7), joint pain/malaise (RD [95% CI] = -11 [-19; -3]; p = 0.009, 11–13 days post-symptom onset), and chest pain (RD [95% CI] = -10 [-19; -0.9]; p = 0.038, day 4), while shortness of breath did not differ significantly between study arms (Figure 2 & Supplementary Table 2). In the non-severe symptom category, there was a significantly lower frequency of a sore throat at 11–13 days post-symptom onset in the active arm (RD [95% CI] = -9 [-18; -0.4]; p = 0.038) and a trend toward a lower frequency of fatigue in the active arm on day 14 (RD [95% CI] = -7 [-16; 1]; p = 0.09) (Figure 2 & Supplementary Table 3). Cough frequency was significantly unbalanced at enrollment with higher frequency in the active arm (p = 0.012) and this persisted until the day 2 visit (p = 0.032). Headache frequency did not differ between study arms. The mITT analysis yielded similar results where fever/chills (day 7), joint pain/malaise (days 7 and 11–13), and chest pain (day 2) were significantly less frequent in the active arm and there was a trend of lower frequency of sore throat (days 7 and 11–13) and fatigue (days 4, 7 and 11–13) in the active arm.

**Figure 3.**
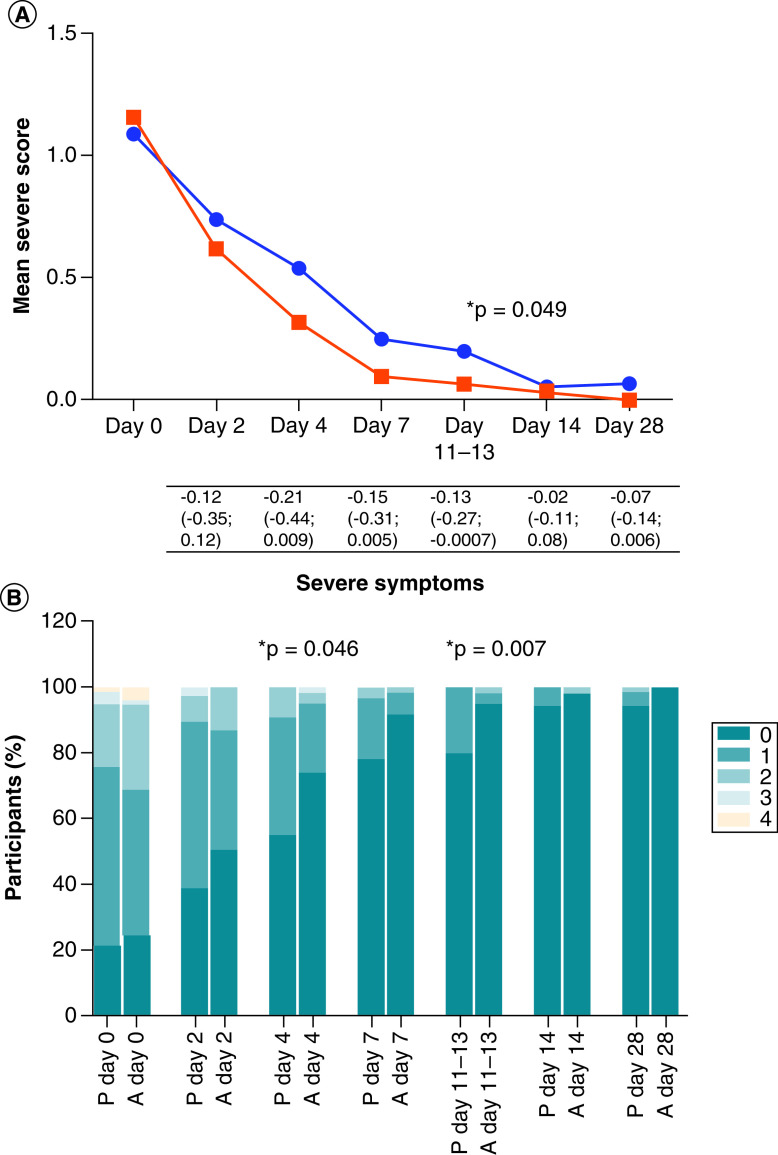

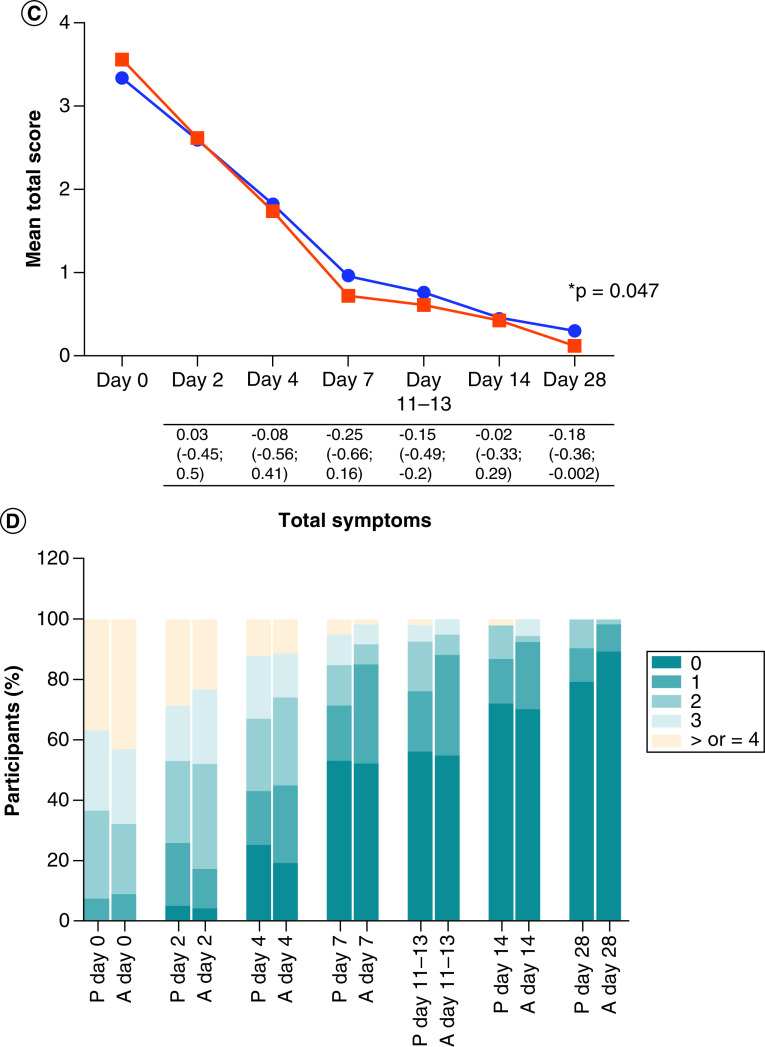
Summative symptom scores. A composite total symptom score (the sum of individual symptoms), composite severe symptom score, and composite non-severe symptom score were calculated for each visit (days 0, 2, 4, 7, 14, and 28 post-enrollment and 11–13 days post-symptom onset). Fever/chills, shortness of breath, chest pain, palpitations, and joint pain/malaise were categorized as severe-type symptoms, while the remaining symptoms (sore throat, cough, fatigue, diarrhea/vomiting, anosmia, dysgeusia, headache, muscular weakness, anxiety/depression, sleep disturbances, cognitive disturbances, and hair loss) were categorized as non-severe. **(A)** Shows the mean severe symptom score at every visit for both placebo and active arms, shown in blue and red, respectively. The severe symptom scores were compared between study arms using the unpaired *T*-test. The mean differences in the severe symptom scores (severe symptom score in the active arm minus that in the placebo arm), with 95% confidence intervals, are shown under the graph. **(B)** Shows the percentage of participants with 0, 1, 2, 3, and 4 severe symptoms at every visit per study arm (P = placebo and A = active), and this was compared between study arms using the Chi-square test. **(C & D)** Represent the total symptoms. P-values less than 0.05 are shown with an asterisk. The total number of participants in each arm at each visit is the same as shown in Figure 1 (intention-to-treat analysis).

The average summative symptom scores at enrollment did not differ significantly between study arms (Table 1), however, the mean severe symptom score was lower in the active arm than the placebo arm for each visit thereafter (Figure 3A & Supplementary Table 4). While this was a trend on day 4 (RD [95% CI] = -0.21 [-0.44; 0.009]; p = 0.06) and day 7 (RD [95% CI] = -0.15 [-0.31; 0.005]; p = 0.058), this reached borderline statistical significance at 11–13 days post-symptom onset (RD [95% CI] = -0.13 [-0.27; -0.0007]; p = 0.049). The symptom score was also presented graphically using stacked bar graphs for ease of visual interpretation. The percentage of participants with 0, 1, 2, 3, and 4 severe symptoms differed significantly between study arms on day 4 (p = 0.046) and days 11–13 (p = 0.007) with fewer symptoms in the active arm, and there was a trend for fewer symptoms in the active arm on day 7 (p = 0.072) (Figure 3B). In a paired analysis comparing the change in symptom score at each visit relative to baseline, the mean change in severe symptom score was greater (a more negative value indicates greater improvement) in the active arm than the placebo arm at each visit, however, this was not statistically significant (Supplementary Table 5). Although the mean total symptom score was slightly lower in the active arm than the placebo arm from day 7 onwards, this was only borderline significant on day 28 (RD [95% CI] = -0.18 [-0.36; -0.002]; p = 0.047) (Figure 3C & Supplementary Table 4) and not significant when comparing the percentage of participants with 0, 1, 2, 3, and ≥4 symptoms (Figure 3D). The paired analysis of the change in total symptom score at each visit relative to baseline showed a similar pattern to the severe score, with a greater mean change in the active arm, although this also was not statistically significant (Supplementary Table 5). The mITT analysis yielded similar results to that of the ITT analysis, with a significantly lower severe symptom score in the active arm on day 7 (p = 0.012) and a tendency for a greater change in total symptom score (i.e., more improvement) in the active arm on day 7 (p = 0.06).

The time to symptom resolution (from the time of symptom onset to clearance of every symptom) could only be analyzed for the 63 participants (40% of the cohort) with complete visits within the visit windows up to day 28. Forty-five participants were observed to achieve complete symptom clearance - 22 in the placebo arm and 23 in the active arm (RD [95% CI] = 5.4 [-16.8; 27.7]; p = 0.9).

In summary, several individual symptoms were less frequent in the active arm and overall symptom scores were lower in the active arm, especially for severe symptoms in the range of days 4 to 11–13, although there was no difference between study arms in time to complete symptom clearance in a smaller subset of the cohort.

### Karnofsky score

The large majority of participants (>96% for all study visits) were in the mild Karnofsky score category, which indicates that they were able to continue with daily activities without assistance. This category is discriminated by three scores – 100 is no symptoms, 90 is any symptoms without extra effort required to perform daily activities, and 80 is any symptoms with extra effort required to perform daily activities. While the mean scores at the follow-up visits were marginally higher in the active arm at all follow-up visits, there was no statistically significant difference between the study arms (Table 2).

**Table 2.** Karnofsky scores.

Visit^†^	Placebo^‡^	Active^‡^	Risk difference^§^	p-value^¶^
Day 0	88.35 (6.87)	88.31 (5.71)		0.97
Day 2	88.55 (9.48)	90.14 (2.71)	1.59 (-0.75; 3.93)	0.18
Day 4	89.85 (11.21)	90.82 (8.02)	0.97 (-2.47; 4.41)	0.58
Day 7	92.71 (11.72)	92.9 (12.06)	0.19 (-4.09; 4.48)	0.93
Day 11–13	93.82 (10.97)	94.33 (8.90)	0.52 (-3.16; 4.19)	0.78
Day 14	95.74 (7.92)	96.30 (7.08)	0.56 (-2.31; 3.42)	0.70
Day 28	97.67 (4.57)	98.48 (3.61)	0.81 (-0.58; 2.21)	0.25
Day 42	96.57 (10.38)	97.79 (4.18)	1.23 (-1.46; 3.91)	0.37

† Visits were on days 0, 2, 4, 7, 14, and 28 post-enrollment and 11–13 days post-symptom onset.

‡ The mean Karnofsky score (standard deviation) at each visit is shown.

§ Risk differences (active arm minus placebo arm), with 95% confidence intervals, are shown.

¶ P-values are from the unpaired *T*-test.

### Viral clearance

Swabs were taken at the clinic visit (11–13 days post-symptom onset) to assess viral clearance. Of the 109 participants who were swabbed within the visit window, SARS-CoV-2 was detected in the swabs of 36/49 (73.5%) participants in the placebo arm and 49/60 (81.7%) participants in the active arm (RD [95% CI] = 8 [-8; 24]; p = 0.3).

### Post-COVID-19 sequelae

Symptoms were present on day 42 in 16/66 (24%) participants in the placebo arm and 15/67 (22%) participants in the active arm (RD [95% CI] = -2 [-16; 13]; p = 0.8) (Table 3). Fatigue, present in 22/31 (71%) individuals with symptoms, was the most common symptom reported. A total of 44 and 28 symptoms were reported in the placebo and active arms, respectively, with mean symptom scores of 0.67 in the placebo arm and 0.42 in the active arm (RD [95% CI] = -0.25 [-0.71; 0.21]; p = 0.28) (Table 3).

**Table 3.** Post-COVID-19 sequelae.

		Placebo (n = 66)	Active (n = 67)	Risk difference^§^	p-value^¶^
Post-COVID-19 sequelae^†^, n (%)	Yes^‡^	16 (24.2)	15 (22.4)	-2 (-16; 13)	0.8
	Cough	4 (6.1)	3 (4.5)	-2 (-9; 6)	0.7
	Shortness of breath	3 (4.6)	0 (0.0)	-5 (-10; 0.5)	0.075
	Joint pain/malaise	6 (9.1)	4 (6.0)	-3 (-12; 6)	0.49
	Fatigue	11 (16.9)	11 (16.4)	-0.5 (-13; 12)	0.94
	Chest pain	3 (4.5)	1 (1.5)	-3 (-9; 3)	0.3
	Loss of smell	2 (3.1)	2 (3.0)	0 (-6; 6)	0.98
	Alteration of taste	4 (6.2)	1 (1.5)	-5 (-11; 2)	0.17
	Headache	1 (1.5)	2 (3.0)	2 (-4; 7)	0.56
	Muscular pain	3 (4.6)	2 (3.0)	-2 (-8; 5)	0.62
	Anxiety/depression	1 (1.5)	1 (1.5)	0 (-4; 4)	0.99
	Sleep disturbance	5 (7.6)	1 (1.5)	-6 (-13; 1)	0.095
	Palpitations	1 (1.5)	0 (0.0)	-2 (-5; 1)	0.31
Total symptoms, mean (SD)		0.67 (1.59)	0.42 (1.02)	-0.25 (-0.71; 0.21)	0.28

† Only symptoms reported on day 42 are shown. Brain fog and hair loss were not reported by any participants on day 42.

‡ Individuals with symptoms on day 42.

§ Risk differences (active arm minus placebo arm), with 95% confidence intervals, are shown.

¶ P-values are from the Chi-square test for categorical variables and the unpaired *T*-test for continuous measures.

SD: Standard deviation.

## Discussion

The therapeutic effect of a hen egg white bovine colostrum mixture in early mild/moderate COVID-19 infection was investigated in this double-blind randomized control study. The main finding was a lower frequency of symptoms, particularly the severe-type symptoms, within the first 2 weeks in the active arm. The symptom most significantly impacted by the intervention was joint pain/malaise, while positive effects were also observed for chest pain, fever, sore throat, and fatigue. Results support that the intervention reduced these symptoms. These findings are likely attributed, in part, to the immunomodulatory effects of proteins in the nutritional supplement. For example, the lactoferrin [51], lysozyme [52,53], and ovotransferrin [36] proteins in the study product, are known to suppress the key pro-inflammatory cytokines interleukin-6 and tumor necrosis factor-alpha, which are also well-known to contribute to pain [54]. Anti-fatigue effects of egg white and milk proteins have previously been reported and linked to anti-oxidant effects [55,56].

The intended primary end point of the study was severe COVID-19, however, contrary to the initial estimates (made in 2020) of progression to severe COVID-19 in symptomatic patients [47,50], only 1 participant out of the 156 enrolled met the definition for severe COVID-19 - the primary end point could therefore not be analyzed. The low rate of severe COVID-19 in the study participants is likely explained by the timing of enrollment. Of the 156 patients enrolled, 45 were enrolled between July and October 2021 during the Delta variant-dominated wave, 88 were enrolled between November 2021 and March 2022 during the Omicron variant (BA.1)-dominated wave, and 23 were enrolled between April and May 2022 during the Omicron variant (BA.4 and BA.5)-dominated wave. Significantly lower disease severity was reported during Omicron waves when compared with waves of previous variants in South Africa [57]. Possible reasons for this include the higher level of immunity acquired by that stage of the pandemic [58] as well as the nature of the Omicron variant, which has a preference for upper airways as opposed to the lower respiratory tract [59].

Although positive effects of the intervention on several symptoms were observed, no significant effect of the intervention on overall symptom duration, Karnofsky score, viral clearance, or post-COVID-19 sequelae was shown. The overall symptom duration analysis (which considered complete clearance of symptoms only) was hampered by the small number of participants who had no missed visits (40% of the cohort). The Karnofsky score measure was initially devised to evaluate the functional status of cancer patients and has largely been used for that purpose [49]. Based on the initial estimates of severe COVID-19, the expectation was that several participants would be more than mildly ill and that the full range of Karnofsky scores would thus be represented. Contrary to this, >96% of the COVID-19 patients in this study were in the mild category of Karnofsky rating where there is limited opportunity to discriminate between and detect smaller differences in the functional status or well-being of symptomatic individuals. While the Karnofsky score and overall symptom duration analyses suffered serious limitations, the viral clearance, and post-COVID-19 sequelae analyses were more robust. There was no positive effect of the intervention on viral clearance, which suggests that the positive impact of the intervention on symptoms was unlikely to be through direct antiviral activity. While bovine lactoferrin has been reported to have a strong direct antiviral effect against a variety of SARS-CoV-2 variants *in vitro* [60], and ingestion of 1 g bovine lactoferrin per day [61], or 0.2 to 1 g bovine lactoferrin per day [62], was reported to significantly reduce SARS-CoV-2 clearance time, the quantity of lactoferrin present in the current supplement (≈ 100 mg daily in two divided amounts) may not have been sufficient to achieve this effect. The presence or absence of post-COVID-19 symptoms was also not significantly affected by the intervention although the overall number of symptoms was on average less in the active arm. This suggests that the earlier discernible benefit of the 5-day intervention on symptoms was attenuated with time.

The greater reduction in symptoms in the first 2 weeks of enrollment in the active arm suggests that the hen egg white bovine colostrum nutritional supplement taken in early COVID-19 infection ameliorates symptoms of the disease. Interestingly, other natural products, for example, vitamin D and honey plus *Nigella sativa* seeds, were reported to significantly ameliorate COVID-19 (including significant effects on symptoms/severity and mortality) with immunomodulatory mechanisms assumed [63,64]. Given the wide range of effects (antioxidant, anti-inflammatory, renin-angiotensin-system inhibitory, antibacterial, antiviral, antifungal) of lactoferrin, lysozyme, and ovotransferrin proteins in the supplement and evidence for the benefit of these proteins in various disease models (e.g., hypertension [65], inflammatory bowel disease [30,66], peritonitis [36], Alzheimer's disease [67]), coupled with the good adherence and safety of the study supplement, there is justification to investigate the therapeutic potential in other disease conditions as well. It is important to note that the quality of the egg white and colostrum products used could affect the outcome. The different dried milk and egg white products tested when selecting products exhibited a range in the quantities of intact lactoferrin, ovotransferrin, and lysozyme proteins (Supplementary Information), influenced by the manufacturing processes. Nevertheless, it is possible to identify dried milk and egg white products with well-preserved functional proteins.

Some additional limitations of this study deserve mention. The sample size was considerably lower than targeted, nevertheless, a signal of the intervention could be detected. The selection of a single time point at 11–13 days post-symptom onset for the follow-up naso-oropharyngeal swab is a limitation of the viral clearance analysis, particularly since the reported average time of viral clearance differs substantially between studies, ranging from <2 weeks to over a month from the time of the first positive test [61,62,68].

## Conclusion

Results support that consumption of a hen egg white bovine colostrum mixture, reconstituted in glycerin BP and distilled water, within a few days of symptom onset lessened symptoms in individuals with mild or moderate COVID-19.

**Summary points**

- Ovotransferrin and lysozyme in hen egg white and lactoferrin in bovine colostrum have immunomodulatory and antiviral properties.
- It was hypothesized that consumption of a hen egg white bovine colostrum mixture in early infection would reduce the risk of progression to severe disease and assist in the recovery of individuals with mild/moderate COVID-19.
- In this double-blind randomized control study, COVID-19-positive individuals at increased risk of severe disease and within 5 days of symptom onset were randomly assigned to consume a hen egg white bovine colostrum mixture (n = 77) or placebo (n = 79) twice daily for 5 days.
- Only 1 participant (active arm) progressed to severe disease.
- The mean protocol-defined severe-type symptom score was significantly lower in the active arm at 11–13 days post-symptom onset (p = 0.049) and there were significantly fewer protocol-defined severe-type symptoms in the active arm on days 4 (p = 0.046) and 11–13 (p = 0.007).
- The individual symptoms that were significantly less frequent in the active arm were chest pain (day 4, p = 0.038), fever/chills (day 7, p = 0.014), joint pain/malaise (days 11–13, p = 0.009), and sore throat (days 11–13, p = 0.038), and there was a tendency for less fatigue in the active arm (day 14, p = 0.09).
- No significant differences in Karnofsky score, viral clearance, or post-COVID-19 sequelae were observed.
- The intervention reduced symptoms in individuals with mild/moderate COVID-19.
- These findings are likely attributed, at least in part, to the immunomodulatory effects of proteins in the nutritional supplement.
