# Supplementary material for: Hen egg white bovine colostrum supplement reduces symptoms of mild/moderate COVID-19: a randomized control trial
Source: Future Sci OA. 2023 Jul 20;9(8):FSO882. doi: 10.2144/fsoa-2023-0024 (PMC10445555; doi:10.2144/fsoa-2023-0024)
Supplement: Supplementary file 1 [file fsoa-09-882-s1.docx]

## Lactoferrin, lysozyme, and ovotransferrin content of the intervention

The rationale for the consumption of egg white and bovine colostrum in COVID-19 infection is based on the reported antimicrobial and immunomodulatory properties of the proteins lactoferrin, ovotransferrin, and lysozyme (reviewed in [1]).

### **1.1 Lactoferrin**

**1.1.1 Lactoferrin content of bovine colostrum: literature**

The concentration of lactoferrin in bovine colostrum varies between breeds and is reported to range from 0.3 to 5 g/L [2-4]. The widely used milk pasteurization treatment of 72 °C - 74 °C for 15 seconds has virtually no effect on lactoferrin structure [5, 6], and spray drying processes result in negligible denaturation of bovine lactoferrin as well as negligible effect on the activity of bovine lactoferrin [7].

**1.1.2 Biolac SA low-heat bovine colostrum powder**

The Biolac bovine colostrum powder was pasteurized and spray dried using low temperatures and was exposed to a maximum of 65 °C during processing. Based on the literature provided above, there would be negligible effect on lactoferrin structure or activity. 14.5 kg Biolac SA bovine colostrum powder is equivalent to 100 L liquid colostrum - this corresponds to a minimum of 2 mg lactoferrin per gram powder based on the literature provided above.

This was confirmed specifically for the Biolac product at the HIV Pathogenesis Programme (HPP) laboratory at the University of KwaZulu-Natal (UKZN) using a commercial sandwich ELISA kit (Bethyl Laboratories, Montgomery, Texas, United States), which is used to detect bovine lactoferrin in milk and only detects intact proteins or large fragments with at least two antibody binding sites [5]. The ELISA procedure was performed according to the manufacturer’s instructions and the final absorbance of the samples was measured at 450 nm using a Victor Nivo^TM^ multimode plate reader (PerkinElmer, Waltham, Massachusetts, United States). A four-parameter standard curve was generated using MyCurveFit (https://mycurvefit.com/) and used to calculate the concentration of lactoferrin in the samples. Samples were assayed in duplicate and averaged. The results showed an average of 2.5 mg (between 2 and 3 mg) detected lactoferrin per gram of Biolac SA colostrum powder (Table 1, MP_F), which is consistent with the literature provided above. The specific batch of colostrum used in the current trial was subsequently tested and had 2.42 gm lactoferrin per gram. Considering the milk products tested, colostrum had the highest lactoferrin content, followed by fresh milk, while medium-heat milk powders had negligible intact lactoferrin.

**Supplementary Table 1. Lactoferrin concentration in milk products determined by sandwich ELISA^a^**

| **Product** | **Category** | **mg/L** | **mg/g** |
| --- | --- | --- | --- |
| Raw milk | Fresh | 91 | - |
| Pasteurised^b^ full cream milk | Fresh | 85 | - |
| MP_A | Medium heat powder | 5 | 0.05 |
| MP_B | Medium heat powder | 2 | 0.02 |
| MP_C | Medium heat powder | Below range | Below range |
| MP_D | Medium heat powder | Below range | Below range |
| MP_E | Low heat powder | 49 | 0.49 |
| MP_F_batch 1 | Low-heat colostrum powder | 295 | 2 |
| MP_F_batch 2 | Low-heat colostrum powder | 449 | 3 |
| MP_F_trial batch | Low-heat colostrum powder | 354 | 2.4 |

^a^ Duplicate absorbance values were within 10 % of each other and the R^2^ values of the standard curves generated were ≥ 0.99.

^b^ Not ultra-high-temperature pasteurization.

###

### **1.2. Lysozyme**

**1.2.1 Lysozyme content of egg white: literature**

The richest accessible source of lysozyme is hen egg white (3.5 % egg white proteins) [8]. Spray drying of egg white results in a small degree of protein denaturation [9, 10]. Lysozyme content is similar to that in raw egg white for most commercial egg white powders [11] and lysozyme activity has been readily detected in spray-dried egg powder [12]. The protein concentration of egg white is 110 mg/ml and lysozyme is 3.5 % of egg white proteins, translating to 3.8 g lysozyme per L of egg white [13].

**1.2.2 EAP sport plus: lysozyme**

1 kg EAP sport plus is made from 8.5 L egg white (manufacturer’s specification), therefore there is 32.3 g lysozyme in the raw egg white used to make 1 kg EAP sport plus [13] – 32 mg lysozyme per g EAP sport plus. To confirm the effect of the processing on lysozyme we measured the enzymatic activity of lysozyme in EAP sport plus compared to raw egg white by the lysoplate method [14], which has previously been used to measure lysozyme activity in clinical samples [15-18]. Briefly, *Micrococcus lysodeikticus* (ATCC 4698, Sigma-Aldrich, St. Louis, Missouri, United States) was suspended uniformly in a small volume of phosphate buffer (0.06 M with 0.02 M NaCl, pH 6.24) and added to molten (60-70 °C) 1% agar, prepared using agarose tablets (TopVision Agarose Tablets, Thermoscientific) and phosphate buffer, to a final concentration of 50 mg of organisms in 100 ml of agar. The agar was poured into petri dishes to a depth of 4 mm and, following solidification, wells of 2 mm in diameter and 4 mm in depth were cut. Ten wells at least 1.5 cm apart were accommodated in a petri dish 85 mm in diameter. Egg white powders were reconstituted according to manufacturers’ instructions, and reconstituted powders as well as raw egg white were diluted 1:7.5 using the phosphate buffer. Standard dilutions (2000, 750, 250, and 75 µg/ml) of purified hen egg white lysozyme (Sigma-Aldrich) were run with each group of test samples. Following the addition of 20 µl of standard or sample to each well, the plates were incubated at room temperature (24-26 °C) for 17 to 19 hours, during which time zones of clearing developed as the result of bacterial lysis. The plates were then photographed using a Chemidoc (Biorad, Hercules, California, United States), and the diameter of each cleared zone was measured from the photograph. A four-parameter standard curve (R^2^ > 0.99) based on the lysozyme standards was used to calculate the lysozyme concentration in the samples, and this value was expressed relative to that in raw egg white, which represented 100 % lysozyme activity.

Lysozyme activity in EAP sport plus (EAP_A in Figure 1A) was comparable to that in raw egg white (93 % activity relative to raw egg white), confirming that the effect of the processes used in the production of EAP sport plus on lysozyme was minimal. The specific batch of EAP sport plus used in the trial was subsequently tested and observed to have 92 % lysozyme activity relative to raw egg white. Other egg white powders tested ranged from 11 – 91 % in lysozyme activity.

**B**

**A**

**Supplementary Figure 1.** **Lysozyme enzymatic activity and ovotransferrin iron-binding activity in egg white products.**

Lysozyme enzymatic activity (panel A) and ovotransferrin iron-binding activity (panel B) in egg white products relative to raw egg white (100 % activity) are shown. EAP_A (EAP sport plus) is the product used in the current trial (the specific batch used is EAP_A_trial). Results are from at least 3 independent experiments. Bars represent the mean and error bars represent the standard deviation from the mean.

### **1.3 Ovotransferrin**

**1.3.1 Ovotransferrin content of egg white: literature**

Spray drying of egg white has no significant effect on egg white ability to limit Salmonella growth, which is primarily attributed to the activity of ovotransferrin, the least heat-stable protein in egg white [9]. However, dry pasteurization conditions may affect ovotransferrin iron-binding activity (as this property requires undenatured protein) [19]. Based on the report of 87 % retention of ovotransferrin iron-binding activity (relative to spray-dried egg white) after hot room treatment at 67 °C for 15 days [19], it is expected that EAP sport plus (with similar hot room treatment conditions) retains this activity. The protein concentration of egg white is 110 mg/ml and lysozyme is 12 % of egg white proteins, translating to 13 g ovotransferrin per L of egg white [13].

**1.3.2 EAP sport plus: ovotransferrin**

1 kg EAP sport plus is made from 8.5 L egg white (manufacturer’s specification), therefore there is 110.5 g ovotransferrin in the raw egg white used to make 1 kg EAP sport plus [13] – 110.5 mg ovotransferrin per g EAP sport plus. Some activities of ovotransferrin require largely intact and undenatured protein, namely iron-binding [19]. Ovotransferrin iron-binding activity in reconstituted egg white products and raw egg white was measured using an iron (III) chloride assay based on previously published methods [19]. Egg white powders were reconstituted to 70 mg/ml in 0.15 M NaCl buffer and raw egg white was diluted to 70 mg/ml in 0.15 M NaCl buffer. Standards were prepared using purified ovotransferrin (Sigma-Aldrich), which was diluted in 0.15 M NaCl buffer to concentrations of 20, 10, 5, 2.5, 1.25, 0.63, and 0.31 mg/ml. In a 96-well plate, 100 µL of diluted sample/standard was mixed with 96 µL of carbonate phosphate citrate buffer (pH 8.5; 0.1 M NaHCO_3_; 0.2 M Na_2_HPO_4_; 0.1 M NaH_2_PO_4_; 0.1 M Na_3_C_6_H_5_O_7_) and 4 µL 20mM FeCl_3_ in 8 replicate wells per sample and duplicate wells for each standard. The plate was incubated in the dark at room temperature following the addition of FeCl_3._ Absorbance at 450 nm was measured on a Victor Nivo^TM^ multimode plate reader before the addition of FeCl_3_ and the change in absorbance at 15 minutes and 1 hour following the addition of FeCl_3_ indicated the iron-binding activity of ovotransferrin. A four-parameter standard curve (R^2^ > 0.99) was used to calculate the ovotransferrin content of the samples, and this was also expressed relative to that in raw egg white.

The iron-binding activity was shown to be retained in EAP sport plus - 73 % relative to raw egg white (EAP_A in Figure 1B). The specific batch of EAP sport plus used in the trial was subsequently tested and observed to have 77 % iron-binding activity relative to raw egg white. Other egg white powders tested ranged in activity from 0 to 70 %.

### **1.4 Summary**

The daily amount of egg white powder and colostrum powder (in 2 divided amounts) is:

24 g EAP sport plus powder containing 0.775 g lysozyme and 2.65 g ovotransferrin

40 g Biolac colostrum powder containing 80-120 mg (average of 100 mg) lactoferrin

Both peptides and intact forms of lactoferrin, lysozyme, and ovotransferrin show desired immunomodulatory and antiviral properties (reviewed in [1]), although certain activities require undenatured/intact protein (*e.g.* iron-binding of ovotransferrin and lactoferrin and enzymatic activity of lysozyme) [19, 20]. The undenatured forms of these proteins are largely preserved in the Biolac colostrum powder and the EAP sport plus.

**1.2 Supplementary symptom tables**

**Supplementary Table 2a. Individual severe symptom frequency (intention-to-treat analysis)**

|  | **A** | **B** | **p** | **A** | **B** | **p** | **A** | **B** | **P** | **A** | **B** | **P** | **A** | **B** | **p** | **A** | **B** | **p** | **A** | **B** | **p** |
| --- | --- | --- | --- | --- | --- | --- | --- | --- | --- | --- | --- | --- | --- | --- | --- | --- | --- | --- | --- | --- | --- |
|  | **D0** | | | **D2** | | | **D4** | | | **D7** | | | **D11-13** | | | **D14** | | | **D28** | | |
| **Fever/chills** |  |  | 0.99 |  |  | 0.97 |  |  | 0.60 |  |  | **0.014** |  |  | 0.58 |  |  | 0.32 |  |  |  |
| Absent | 35  (44) | 34  (44) |  | 50  (65) | 45  (65) |  | 55  (82) | 53  (85) |  | **52**  **(87)** | **60**  **(98)** |  | 52  (94) | 58  (97) |  | 54  (100) | 53  (98) |  | 73  (100) | 66  (100) |  |
| Present | 44  (56) | 43  (56) |  | 27  (35) | 24  (35) |  | 12  (18) | 9  (15) |  | **8**  **(13)** | **1**  **(2)** |  | 3  (6) | 2  (3) |  |  | 1  (2) |  |  |  |  |
| **Shortness of breath** |  |  | 0.95 |  |  | 0.93 |  |  | 0.82 |  |  | 0.32 |  |  | 0.34 |  |  | 0.32 |  |  |  |
| Absent | 67  (85) | 65  (84) |  | 70  (91) | 63  (91) |  | 62  (92) | 58  (93) |  | 60  (100) | 60  (98) |  | 55  (100) | 59  (98) |  | 54  (100) | 53  (98) |  | 73  (100) | 66  (100) |  |
| Present | 12  (15) | 12  (16) |  | 7  (9) | 6  (9) |  | 5  (8) | 4  (7) |  |  | 1  (2) |  |  | 1  (2) |  |  | 1  (2) |  |  |  |  |
| **Joint pain/malaise** |  |  | 0.21 |  |  | 0.83 |  |  | 0.22 |  |  | 0.14 |  |  | **0.009** |  |  | 0.079 |  |  | 0.054 |
| Absent | 65  (82) | 57  (74) |  | 66  (86) | 60  (87) |  | 57  (85) | 57  (92) |  | 54  (90) | 59  (97) |  | **49**  **(89)** | **60**  **(100)** |  | 51  (94) | 54  (100) |  | 69  (94) | 66  (100) |  |
| Present | 14  (18) | 20  (26) |  | 11  (14) | 9  (13) |  | 10  (15) | 5  (8) |  | 6  (10) | 2  (3) |  | **6**  **(11)** |  |  | 3  (6) |  |  | 4  (6) |  |  |
| **Chest pain** |  |  | 0.90 |  |  | 0.059 |  |  | **0.038** |  |  | 0.57 |  |  | 0.51 |  |  |  |  |  |  |
| Absent | 64  (81) | 63  (82) |  | 65  (84) | 65  (94) |  | **58**  **(87)** | **60**  **(97)** |  | 59  (98) | 59  (97) |  | 53  (96) | 59  (98) |  | 54  (100) | 54  (100) |  | 72  (99) | 66  (100) | 0.34 |
| Present | 15  (19) | 14  (18) |  | 12  (16) | 4  (6) |  | **9**  **(13)** | **2**  **(3)** |  | 1  (2) | 2  (3) |  | 2  (4) | 1  (2) |  |  |  |  | 1  (1) |  |  |
| **Palpitations** |  |  | 0.32 |  |  |  |  |  |  |  |  |  |  |  |  |  |  |  |  |  |  |
| Absent | 78  (99) | 77  (100) |  | 77  (100) | 69  (100) |  | 67  (100) | 62  (100) |  | 60  (100) | 61  (100) |  | 55  (100) | 60  (100) |  | 54  (100) | 54  (100) |  | 73  (100) | 66  (100) |  |
| Present | 1  (1) |  |  |  |  |  |  |  |  |  |  |  |  |  |  |  |  |  |  |  |  |

A – placebo arm; B – active arm; p – p-value; D0, D2, D4, D7, D14, D28 – 0, 2, 4, 7, 14, and 28 days post-enrollment; D11-13 – 11-13 days post-symptom onset.

The number of participants is shown with percentages in brackets. Significant differences (Chi-square test) in symptom frequency between arms are shown in red.

**Supplementary Table 2b. Individual severe symptom frequency: risk estimates and confidence intervals**

|  | **RR** | **RD** | **RR** | **RD** | **RR** | **RD** | **RR** | **RD** | **RR** | **RD** | **RR** | **RD** |
| --- | --- | --- | --- | --- | --- | --- | --- | --- | --- | --- | --- | --- |
|  | **D2** | | **D4** | | **D7** | | **D11-13** | | **D14** | | **D28** | |
| **Fever/chills** | 0.99  (0.64; 1.55) | -0.3  (-16; 15) | 0.81  (0.37; 1.79) | -3  (-16; 9) | **0.12**  **(0.02; 0.95)** | **-12**  **(-21; -3)** | 0.61  (0.11 – 3.52) | -2  (-10; 5) | 0 | 2  (-2; 5) | N/A | 0  (0; 0) |
| **Shortness of breath** | 0.96  (0.34; 2.71) | -0.4  (-10; 9) | 0.86  (0.24; 3.07) | -1  (-10; 8) | 0 | 2  (-2; 5) | 0 | 2  (-2; 5) | 0 | 2  (-2; 5) | N/A | 0  (0; 0) |
| **Joint pain/malaise** | 0.91  (0.40; 2.07) | -1  (-12; 10) | 0.54  (0.2; 1.49) | -7  (-18; 4) | 0.33  (0.07; 1.56) | -7  (-16; 2) | **0** | **-11**  **(-19; -3)** | 0 | -6  (-12; 0.6) | 0 | -5  (-11; -0.3) |
| **Chest pain** | 0.37  (0.13; 1.10) | -10  (-20; 0.01) | **0.24**  **(0.05; 1.07)** | **-10**  **(-19; -0.9)** | 1.97  (0.18; 21.13) | 2  (-4; 7) | 0.46  (0.04 – 4.91) | -2  (-8; 4) | N/A | 0  (0; 0) | 0 | -1  (-4; 1) |
| **Palpitations** | N/A | 0  (0; 0) | N/A | 0  (0; 0) | N/A | 0  (0; 0) | N/A | 0  (0; 0) | N/A | 0  (0; 0) | N/A | 0  (0; 0) |

RR – risk ratio (active arm divided by placebo arm) with 95 % confidence intervals; RD – risk difference (% with symptom in the active arm minus % with symptom in the placebo arm) with 95 % confidence intervals; N/A – not applicable as no ratio could be calculated; D0, D2, D4, D7, D14, D28 – 0, 2, 4, 7, 14, and 28 days post-enrollment; D11-13 – 11-13 days post-symptom onset.

Significant differences (Chi-square test) between the active and placebo arms are shown in red.

**Supplementary Table 3a. Individual non-severe symptom frequency (intention-to-treat analysis)**

|  | **A** | **B** | **P** | **A** | **B** | **P** | **A** | **B** | **p** | **A** | **B** | **P** | **A** | **B** | **p** | **A** | **B** | **p** | **A** | **B** | **p** |
| --- | --- | --- | --- | --- | --- | --- | --- | --- | --- | --- | --- | --- | --- | --- | --- | --- | --- | --- | --- | --- | --- |
|  | **D0** | | | **D2** | | | **D4** | | | **D7** | | | **D11-13** | | | **D14** | | | **D28** | | |
| **Sore throat** |  |  | 0.89 |  |  | 0.50 |  |  | 0.81 |  |  | 0.14 |  |  | **0.038** |  |  | 1.00 |  |  | 0.18 |
| Absent | 45  (57) | 43  (56) |  | 51  (66) | 42  (61) |  | 52  (78) | 47  (76) |  | 54  (90) | 59  (97) |  | **49**  **(89)** | **59**  **(98)** |  | 52  (96) | 52  (96) |  | 71  (97) | 66  (100) |  |
| Present | 34  (43) | 34  (44) |  | 26  (34) | 27  (39) |  | 15  (22) | 15  (24) |  | 6  (10) | 2  (3) |  | **6**  **(11)** | **1**  **(2)** |  | 2  (4) | 2  (4) |  | 2  (3) |  |  |
| **Cough** |  |  | **0.012** |  |  | **0.032** |  |  | 0.12 |  |  | 0.60 |  |  | 0.20 |  |  | 1.00 |  |  | 0.25 |
| Absent | **26**  **(33)** | **12**  **(16)** |  | **28**  **(36)** | **14**  **(20)** |  | 35  (52) | 24  (39) |  | 42  (70) | 40  (66) |  | 45  (82) | 43  (76) |  | 44  (81) | 44  (81) |  | 66  (90) | 63  (95) |  |
| Present | **53**  **(67)** | **65**  **(84)** |  | **49**  **(64)** | **55**  **(80)** |  | 32  (48) | 38  (61) |  | 18  (30) | 21  (34) |  | 10  (18) | 17  (24) |  | 10  (19) | 10  (19) |  | 7  (10) | 3  (5) |  |
| **Fatigue** |  |  | 0.78 |  |  | 0.26 |  |  | 0.12 |  |  | 0.14 |  |  | 0.24 |  |  | 0.093 |  |  | 0.30 |
| Absent | 57  (72) | 54  (70) |  | 59  (77) | 58  (84) |  | 54  (81) | 56  (90) |  | 54  (90) | 59  (97) |  | 49  (89) | 57  (95) |  | 49  (91) | 53  (98) |  | 68  (93) | 64  (97) |  |
| Present | 22  (28) | 23  (30) |  | 18  (23) | 11  (16) |  | 13  (19) | 6  (10) |  | 6  (10) | 2  (3) |  | 6  (11) | 3  (5) |  | 5  (9) | 1  (2) |  | 5  (7) | 2  (3) |  |
| **Diarrhea/vomiting** |  |  | 0.54 |  |  | 0.31 |  |  | 0.20 |  |  | 0.99 |  |  | 0.61 |  |  | 1.00 |  |  |  |
| Absent | 73  (92) | 69  (90) |  | 69  (90) | 65  (94) |  | 63  (94) | 61  (98) |  | 59  (98) | 60  (98) |  | 54  (98) | 58  (97) |  | 53  (98) | 53  (98) |  | 73  (100) | 66  (100) |  |
| Present | 6  (8) | 8  (10) |  | 8  (10) | 4  (6) |  | 4  (6) | 1  (2) |  | 1  (2) | 1  (2) |  | 1  (2) | 2  (3) |  | 1  (2) | 1  (2) |  |  |  |  |
| **Anosmia** |  |  | 0.61 |  |  | 0.45 |  |  | 0.64 |  |  | 0.66 |  |  | 0.17 |  |  |  |  |  | 0.34 |
| Absent | 71  (90) | 71  (92) |  | 70  (91) | 65  (94) |  | 63  (94) | 57  (92) |  | 58  (97) | 58  (95) |  | 55  (100) | 58  (97) |  | 54  (100) | 54  (100) |  | 72  (99) | 66  (100) |  |
| Present | 8  (10) | 6  (8) |  | 7  (9) | 4  (6) |  | 4  (6) | 5  (8) |  | 2  (3) | 3  (5) |  | 0  (0) | 2  (3) |  |  |  |  | 1  (1) |  |  |
| **Dysgeusia** |  |  | 0.27 |  |  | 0.44 |  |  | 0.31 |  |  | 0.68 |  |  | 0.58 |  |  | 0.56 |  |  | 0.92 |
| Absent | 73  (92) | 67  (87) |  | 71  (92) | 61  (88) |  | 62  (92) | 54  (87) |  | 56  (93) | 58  (95) |  | 52  (94) | 58  (97) |  | 53  (98) | 52  (96) |  | 71  (97) | 64  (97) |  |
| Present | 6  (8) | 10  (13) |  | 6  (8) | 8  (12) |  | 5  (8) | 8  (13) |  | 4  (7) | 3  (5) |  | 3  (6) | 2  (3) |  | 1  (2) | 2  (4) |  | 2  (3) | 2  (3) |  |
| **Headache** |  |  | 1.00 |  |  | 0.39 |  |  | 0.38 |  |  | 0.73 |  |  | 0.83 |  |  | 0.17 |  |  | 0.29 |
| Absent | 40  (51) | 39  (51) |  | 51  (66) | 41  (59) |  | 56  (84) | 48  (77) |  | 54  (90) | 56  (92) |  | 51  (93) | 55  (92) |  | 53  (98) | 50  (95) |  | 73  (100) | 65  (98) |  |
| Present | 39  (49) | 38  (49) |  | 26  (34) | 28  (41) |  | 11  (16) | 14  (23) |  | 6  (10) | 5  (8) |  | 4  (7) | 5  (8) |  | 1  (2) | 4  (5) |  |  | 1  (2) |  |
| **Muscular weakness** |  |  | **0.045** |  |  | 0.18 |  |  | 0.33 |  |  |  |  |  | 0.95 |  |  | 1.00 |  |  |  |
| Absent | **75**  **(95)** | **77**  **(100)** |  | 75  (97) | 69  (100) |  | 66  (98) | 62  (100) |  | 60  (100) | 61  (100) |  | 54  (98) | 59  (98) |  | 53  (98) | 53  (98) |  | 73  (100) | 66  (100) |  |
| Present | **4**  **(5)** |  |  | 2  (3) |  |  | 1  (2) |  |  |  |  |  | 1  (2) | 1  (2) |  | 1  (2) | 1  (2) |  |  |  |  |
| **Anxiety/depression** |  |  | 0.16 |  |  | 0.34 |  |  | 0.96 |  |  | 0.32 |  |  |  |  |  |  |  |  |  |
| Absent | 77  (97) | 77  (100) |  | 76  (99) | 69  (100) |  | 66  (98) | 61  (98) |  | 60  (100) | 60  (98) |  | 55  (100) | 60  (100) |  | 54  (100) | 54  (100) |  | 73  (100) | 66  (100) |  |
| Present | 2  (3) |  |  | 1  (1) |  |  | 1  (2) | 1  (2) |  |  | 1  (2) |  |  |  |  |  |  |  |  |  |  |
| **Sleep disturbance** |  |  | 0.084 |  |  |  |  |  |  |  |  |  |  |  |  |  |  |  |  |  |  |
| Absent | 76  (96) | 77  (100) |  | 77  (100) | 69  (100) |  | 67  (100) | 62  (100) |  | 60  (100) | 61  (100) |  | 55  (100) | 60  (100) |  | 54  (100) | 54  (100) |  | 73  (100) | 66  (100) |  |
| Present | 3  (4) |  |  |  |  |  |  |  |  |  |  |  |  |  |  |  |  |  |  |  |  |
| **Cognitive disturbance** |  |  | 0.99 |  |  | 0.29 |  |  |  |  |  |  |  |  |  |  |  |  |  |  |  |
| Absent | 78  (99) | 76  (99) |  | 77  (100) | 68  (99) |  | 67  (100) | 62  (100) |  | 60  (100) | 61  (100) |  | 55  (100) | 60  (100) |  | 54  (100) | 54  (100) |  | 73  (100) | 66  (100) |  |
| Present | 1  (1) | 1  (1) |  |  | 1  (1) |  |  |  |  |  |  |  |  |  |  |  |  |  |  |  |  |
| **Hair loss** |  |  |  |  |  |  |  |  |  |  |  |  |  |  |  |  |  |  |  |  |  |
| Absent | 79  (100) | 77  (100) |  | 77  (100) | 69  (100) |  | 67  (100) | 62  (100) |  | 60  (100) | 61  (100) |  | 55  (100) | 60  (100) |  | 54  (100) | 54  (100) |  | 73  (100) | 66  (100) |  |

A – placebo arm; B – active arm; p – p-value; D0, D2, D4, D7, D14, D28 – 0, 2, 4, 7, 14, and 28 days post-enrollment; D11-13 – 11-13 days post-symptom onset.

The number of participants is shown with percentages in brackets. Significant differences (Chi-square test in symptom frequency between arms are shown in red.

**Supplementary Table 3b. Individual non-severe symptom frequency: risk estimates and confidence intervals**

|  | **RR** | **RD** | **RR** | **RD** | **RR** | **RD** | **RR** | **RD** | **RR** | **RD** | **RR** | **RD** |
| --- | --- | --- | --- | --- | --- | --- | --- | --- | --- | --- | --- | --- |
|  | **D2** | | **D4** | | **D7** | | **D11-13** | | **D14** | | **D28** | |
| **Sore throat** | 1.16  (0.75 – 1.78) | 5  (-10; 21) | 1.08  (0.58 – 2.02) | 2  (-13; 16) | 0.33  (0.07 – 1.56) | -7  (-16; 2) | **0.15**  **(0.02 – 1.23)** | **-9**  **(-18; -0.4)** | 1  (0.15 – 6.84) | 0  (-7; 7) | 0 | -3  (-6; 1) |
| **Cough** | **1.25**  **(1.02 – 1.54)** | **16**  **(2; 30)** | 1.28  (0.93 – 1.77) | 14  (-4; 31) | 1.15  (0.68 – 1.93) | 4  (-12; 21) | 1.56  (0.78 – 3.11) | 10  (-5; 25) | 1  (0.45 – 2.21) | 0  (-15; 15) | 0.47  (0.13 – 1.76) | -5  (-13; 3) |
| **Fatigue** | 0.68  (0.35 – 1.34) | -7  (-20; 5) | 0.5  (0.2 – 1.23) | -10  (-22; 2) | 0.33  (0.07 – 1.56) | -7  (-16; 2) | 0.46  (0.12 – 1.74) | -6  (-16; 4) | 0.2  (0.02 – 1.66) | -7  (-16; 1) | 0.44  (0.09 – 2.2) | -4  (-11; 3) |
| **Diarrhea/vomiting** | 0.56  (0.18 -1.78) | -5  (-13; 4) | 0.27  (0.03 – 2.35) | -4  (-11; 2) | 0.98  (0.06 – 15.37) | 0  (-5; 5) | 1.83  (0.17 – 19.66) | 2  (-4; 7) | 1  (0.06 – 15.58) | 0  (-5; 5) | N/A | 0  (0; 0) |
| **Anosmia** | 0.64  (0.20 – 2.09) | -3  (-12; 5) | 1.35  (0.38 – 4.8) | 2  (-7; 11) | 1.48  (0.26 – 8.52) | 2  (-5; 9) | N/A | 3  (-1; 8) | N/A | 0  (0; 0) | 0 | -1  (-4; 1) |
| **Dysgeusia** | 1.49  (0.54 – 4.07) | 4  (-6; 13) | 1.73  (0.6 – 5.0) | 5  (-5; 16) | 0.74  (0.17 – 3.16) | -2  (-10; 7) | 0.61  (0.11 – 3.52) | -2  (-10; 5) | 0.2  (0.19 – 21.41) | 2  (-4; 8) | 1.11  (0.16 – 7.63) | 0.3  (-5; 6) |
| **Headache** | 1.20  (0.79 – 1.84) | 7  (-9; 22) | 1.38  (0.68 – 2.8) | 6  (-8; 20) | 0.82  (0.26 – 2.54) | -2  (-12; 8) | 1.15  (0.32 – 4.05) | 1  (-9; 11) | 4  (0.46 – 34.64) | 6  (-2; 13) | N/A | 2  (-1; 4) |
| **Muscular weakness** | 0 | -3  (-6; 1) | 0 | -1  (-4; 1) | N/A | 0  (0; 0) | 0.92  (0.06 – 14.3) | 0  (-5; 5) | 1  (0.06 – 15.58) | 0  (-5; 5) | N/A | 0  (0; 0) |
| **Anxiety/depression** | 0 | -1  (-4; 1) | 1.08  (0.07 – 16.91) | 0  (-4; 4) | N/A | 2  (-2; 5) | N/A | 0  (0; 0) | N/A | 0  (0; 0) | N/A | 0  (0; 0) |
| **Sleep disturbance** | N/A | 0  (0; 0) | N/A | 0  (0; 0) | N/A | 0  (0; 0) | N/A | 0  (0; 0) | N/A | 0  (0; 0) | N/A | 0  (0; 0) |
| **Cognitive disturbance** | N/A | 1  (-1; 4) | N/A | 0  (0; 0) | N/A | 0  (0; 0) | N/A | 0  (0; 0) | N/A | 0  (0; 0) | N/A | 0  (0; 0) |
| **Hair loss** | N/A | 0  (0; 0) | N/A | 0  (0; 0) | N/A | 0  (0; 0) | N/A | 0  (0; 0) | N/A | 0  (0; 0) | N/A | 0  (0; 0) |

RR – risk ratio (active arm divided by placebo arm) with 95 % confidence intervals; RD – risk difference (% with symptom in the active arm minus % with symptom in the placebo arm) with 95 % confidence intervals; N/A – not applicable as no ratio could be calculated; D0, D2, D4, D7, D14, D28 – 0, 2, 4, 7, 14, and 28 days post-enrollment; D11-13 – 11-13 days post-symptom onset.

Significant differences (Chi-square test) between the active and placebo arms are shown in red.

**Supplementary Table 4. Symptom scores (intention-to-treat analysis)**

|  | **Visit** | **Placebo ^a^** | **Active ^a^** | **Risk difference ^b^** | **p-value ^c^** |
| --- | --- | --- | --- | --- | --- |
| **Severe score** | D0 | 1.09 (0.82) | 1.16 (0.95) |  | 0.64 |
|  | D2 | 0.74 (0.71) | 0.62 (0.71) | -0.12 (-0.35; 0.12) | 0.32 |
|  | D4 | 0.54 (0.66) | 0.32 (0.62) | -0.21 (-0.44; 0.009) | 0.06 |
|  | D7 | 0.25 (0.51) | 0.098 (0.35) | -0.15 (-0.31; 0.005) | 0.058 |
|  | D11-13 | 0.2 (0.4) | 0.067 (0.31) | -0.13 (-0.27; -0.0007) | **0.049** |
|  | D14 | 0.055 (0.23) | 0.037 (0.27) | -0.02 (-0.11; 0.08) | 0.70 |
|  | D28 | 0.068 (0.3) | 0 (0) | -0.07 (-0.14; 0.006) | 0.07 |
| **Non-severe score** | D0 | 2.25 (1.44) | 2.4 (1.34) |  | 0.5 |
|  | D2 | 1.86 (1.23) | 2 (1.06) | 0.14 (-0.23; 0.52) | 0.46 |
|  | D4 | 1.28 (1.1) | 1.42 (1.12) | 0.14 (-0.25; 0.52) | 0.49 |
|  | D7 | 0.72 (0.99) | 0.62 (0.82) | -0.09 (-0.42; 0.23) | 0.57 |
|  | D11-13 | 0.56 (0.88) | 0.55 (0.72) | -0.01 (-0.31; 0.28) | 0.93 |
|  | D14 | 0.39 (0.76) | 0.39 (0.71) | 0 (-0.28; 0.28) | 1 |
|  | D28 | 0.23 (0.54) | 0.12 (0.37) | -0.11 (-0.27; 0.05) | 0.16 |
| **Total score** | D0 | 3.34 (1.8) | 3.56 (1.86) |  | 0.46 |
|  | D2 | 2.6 (1.52) | 2.62 (1.35) | 0.03 (-0.45; 0.5) | 0.91 |
|  | D4 | 1.82 (1.48) | 1.74 (1.29) | -0.08 (-0.56; 0.41) | 0.75 |
|  | D7 | 0.97 (1.29) | 0.72 (0.97) | -0.25 (-0.66; 0.16) | 0.24 |
|  | D11-13 | 0.76 (1.04) | 0.62 (0.83) | -0.15 (-0.49; 0.2) | 0.40 |
|  | D14 | 0.44 (0.84) | 0.43 (0.79) | -0.02 (-0.33; 0.29) | 0.91 |
|  | D28 | 0.30 (0.64) | 0.12 (0.37) | -0.18 (-0.36; -0.002) | **0.047** |

D0, D2, D4, D7, D14, D28 – 0, 2, 4, 7, 14, and 28 days post-enrollment; D11-13 – 11-13 days post-symptom onset.

^a^ The mean symptom score (standard deviation) at each visit is shown.

^b^ Risk difference (active arm minus placebo arm), with 95 % confidence intervals, is shown.

^c^ Significant differences (unpaired T-test) in symptom frequency between arms are shown in red.

**Supplementary Table 5. Change in symptom scores (intention-to-treat analysis)**

|  | **Visit** | **Placebo ^a^** | **Active ^a^** | **Risk difference ^b^** | **p-value ^c^** |
| --- | --- | --- | --- | --- | --- |
| **Severe score** | D2 | -0.35 (0.7) | -0.54 (0.85) | -0.19 (-0.44; 0.07) | 0.15 |
|  | D4 | -0.61 (0.8) | -0.84 (0.99) | -0.23 (-0.54; 0.09) | 0.15 |
|  | D7 | -0.85 (0.8) | -1 (0.97) | -0.15 (-0.47; 0.17) | 0.35 |
|  | D11-13 | -0.85 (0.73) | -1.05 (0.85) | -0.2 (-0.49; 0.1) | 0.19 |
|  | D14 | -1 (0.8) | -1.07 (0.84) | -0.07 (-0.39; 0.24) | 0.64 |
|  | D28 | -1.05 (0.81) | -1.15 (0.95) | -0.1 (-0.39; 0.2) | 0.52 |
| **Non-severe score** | D2 | -0.42 (1.14) | -0.46 (0.93) | -0.05 (-0.39; 0.29) | 0.78 |
|  | D4 | -0.99 (1.38) | -0.97 (1.39) | 0.02 (-0.46; 0.5) | 0.94 |
|  | D7 | -1.35 (1.33) | -1.77 (1.52) | -0.42 (-0.93; 0.09) | 0.11 |
|  | D11-13 | -1.67 (1.48) | -1.87 (1.53) | -0.19 (-0.75; 0.36) | 0.49 |
|  | D14 | -1.94 (1.58) | -2.06 (1.48) | -0.11 (-0.7; 0.47) | 0.71 |
|  | D28 | -2.05 (1.39) | -2.35 (1.34) | -0.29 (-0.75; 0.17) | 0.21 |
| **Total score** | D2 | -0.77 (1.62) | -1 (1.47) | -0.23 (-0.74; 0.27) | 0.36 |
|  | D4 | -1.6 (1.84) | -1.81 (2.02) | -0.21 (-0.88; 0.46) | 0.54 |
|  | D7 | -2.2 (1.63) | -2.77 (2.08) | -0.57 (-1.24; 0.1) | 0.096 |
|  | D11-13 | -2.53 (1.68) | -2.92 (1.96) | -0.39 (-1.07; 0.29) | 0.26 |
|  | D14 | -2.94 (1.9) | -3.13 (1.8) | -0.19 (-0.89; 0.52) | 0.60 |
|  | D28 | -3.11 (1.74) | -3.5 (1.79) | -0.39 (-0.98; 0.2) | 0.19 |

D2, D4, D7, D14, D28 – 0, 2, 4, 7, 14, and 28 days post-enrollment; D11-13 – 11-13 days post-symptom onset

^a^ The symptom score at each visit minus the symptom score at enrollment is shown. The mean change in symptom score (standard deviation) is shown.

^b^ Risk difference (active arm minus placebo arm), i.e. mean difference between arms in the change in symptom scores, with 95 % confidence intervals, is shown.

^c^ The unpaired T-test was used to test for significant differences between the study arms.

**References**

1. Mann JK, Ndung'u T. The potential of lactoferrin, ovotransferrin and lysozyme as antiviral and immune-modulating agents in COVID-19*.* *Future Virol.*  doi: 10.2217/fvl-2020-0170 (2020).

2. Mcgrath BA, Fox PF, Mcsweeney PLH, Kelly AL. Composition and properties of bovine colostrum: a review. *Dairy Sci. Technol.* 96, 133-158 (2015).

3. Tsuji S, Hirata Y, Mukai F, Ohtagaki S. Comparison of Lactoferrin Content in Colostrum Between Different Cattle Breeds. *J. Dairy Sci.* 73(1), 125-128 (1990).

4. Yoshida S, Wei Z, Shinmura Y, Fukunaga N. Separation of Lactoferrin-A and -B From Bovine Colostrum. *J. Dairy Sci.* 83(10), 2211-2215 (2000).

5. Dupont D, Arnould C, Rolet-Repecaud O *et al*. Determination of bovine lactoferrin concentrations in cheese with specific monoclonal antibodies. *Int. Dairy J.* 16(9), 1081-1087 (2006).

6. Sanchez L, Peiro JM, Castillo H, Perez MD, Ena JM, Calvo M. Kinetic Parameters for Denaturation of Bovine Milk Lactoferrin. *J. Food Sci.* 57(4), 873-879 (1992).

7. Wang B, Timilsena YP, Blanch E, Adhikari B. Characteristics of bovine lactoferrin powders produced through spray and freeze drying processes*.* *Int. J. Biol. Macromol.* 95, 985-994 (2017).

8. Abeyrathne EDNS, Lee HY, Ahn DU. Egg white proteins and their potential use in food processing or as nutraceutical and pharmaceutical agents—A review. *Poult. Sci.* 92(12), 3292-3299 (2013).

9. Baron F, Gautier M, Brulé G. Rapid growth of Salmonella enteritidis in egg white reconstituted from industrial egg white powder*.* *J. Food Prot.* 62(6), 585-591 (1999).

10. Katekhong W, Charoenrein S. Influence of spray drying temperatures and storage conditions on physical and functional properties of dried egg white*.* *Dry. Technol.* 36(2), 169-177 (2018).

11. Weth F, Schroeder T, Buxtorf UP. Determination of lysozyme content in eggs and egg products using SDS-gel electrophoresis*.* *Z. Lebensm Unters Forsch* 187(6), 541-545 (1988).

12. Hartsell SE. Lysozyme activity of rehydrated, spray-dried, whole-egg powder. *Food Res.* 13(2), 136-142 (1948).

13. Réhault-Godbert S, Guyot N, Nys Y. The Golden Egg: Nutritional Value, Bioactivities, and Emerging Benefits for Human Health. *Nutrients* 11(3), E684 (2019).

14. Osserman EF, Lawlor DP. Serum and urinary lysozyme (muramidase) in monocytic and monomyelocytic leukemia*.* *J. Exp. Med.* 124(5), 921-952 (1966).

15. Etches PC, Leahy F, Harris D, Baum JD. Lysozyme in the tears of newborn babies*.* *Arch. Dis. Child* 54(3), 218-221 (1979).

16. Maeda K, Ito K, Yamaguchi N. A simple lysoplate method of lysozyme determination with samples dried on filter paper*.* *Clin. Chim. Acta* 100(2), 175-181 (1980).

17. Jenzano JW, Hogan SL, Lundblad RL. Factors influencing measurement of human salivary lysozyme in lysoplate and turbidimetric assays*.* *J. Clin. Microbiol.* 24(6), 963-967 (1986).

18. Hankiewicz J, Swierczek E. Lysozyme in human body fluids. *Clin. Chim. Acta* 57(3), 205-209 (1974).

19. Baron F, Nau F, Guérin-Dubiard C, Gonnet F, Dubois J, Gautier M. Effect of dry heating on the microbiological quality, functional properties, and natural bacteriostatic ability of egg white after reconstitution. *J. Food Prot.* 66(5), 825-832 (2003).

20. Aminlari L, Hashemi MM, Aminlari M. Modified Lysozymes as Novel Broad Spectrum Natural Antimicrobial Agents in Foods. *J. Food Sci.* 79(6), 1077-1090 (2014).
